# Effect of the Growth Assessment Protocol on the DEtection of Small for GestatioNal age fetus: process evaluation from the DESiGN cluster randomised trial

**DOI:** 10.1186/s13012-022-01228-1

**Published:** 2022-09-05

**Authors:** Sophie Relph, Kirstie Coxon, Matias C. Vieira, Andrew Copas, Andrew Healey, Alessandro Alagna, Annette Briley, Mark Johnson, Deborah A. Lawlor, Christoph Lees, Neil Marlow, Lesley McCowan, Jessica McMicking, Louise Page, Donald Peebles, Andrew Shennan, Baskaran Thilaganathan, Asma Khalil, Dharmintra Pasupathy, Jane Sandall, on behalf of the DESiGN Collaborative Group, Spyros Bakalis, Claire Rozette, Marcelo Canda, Simona Cicero, Olayinka Akinfenwa, Philippa Cox, Lisa Giacometti, Elisabeth Peregrine, Lyndsey Smith, Sam Page, Deepa Janga, Sandra Essien, Renata Hutt, Yaa Acheampong, Bonnie Trinder, Louise Rimell, Janet Cresswell, Sarah Petty, Bini Ajay, Hannah O’Donnell, Emma Wayman, Mandish Dhanjal, Muna Noori, Elisa Iaschi, Raffaele Napolitano, Iris Tsikimi, Rachel Das, Fiona Ghalustians, Francesca Hanks, Laura Camarasa, Hiran Samarage, Stephen Hiles, Anna David, David Howe, Nadine Seward, Elizabeth Allen, Jillian Francis

**Affiliations:** 1 Department of Women and Children’s Health, Faculty of Life Sciences and Medicine, School of Life Course Sciences, Women’s Health Academic Centre KHP, King’s College London, 10th Floor North Wing, St. Thomas’ Hospital, Westminster Bridge Road, London, SE1 7EH UK; 2 Department of Midwifery, Faculty of Health, Social Care and Education, Kingston and St. George’s Universities, Kenry House, Kingston Hill, London, KT2 7LB UK; 3 Department of Obstetrics and Gynaecology, School of Medical Sciences, University of Campinas (UNICAMP), Campinas, SP 13083-881 Brazil; 4 Centre for Pragmatic Global Health Trials, Institute for Global Health, University College London, Gower Street, London, WC1E 6BT UK; 5 Centre for Implementation Science and King’s Health Economics, Health Services and Population Research Department, Institute of Psychiatry, Psychology & Neuroscience at King’s College London, The David Goldberg Centre, London, SE5 8AF UK; 6 The Guy’s & St Thomas’ Charity, 9 King’s Head Yard, London, SE1 1NA UK; 7 Caring Futures Institute Flinders University and North Adelaide Local Health Network, Adelaide, SA 5042 Australia; 8 Department of Surgery and Cancer, Imperial College London, Kensington, London, SW7 2AZ UK; 9 Bristol NIHR Biomedical Research Centre, Bristol, BS8 2BL UK; 10 Medical Research Council Integrative Epidemiology Unit at the University of Bristol, Bristol, BS8 2BL UK; 11 Population Health Science, Bristol Medical School, University of Bristol, Bristol, BS8 2BL UK; 12 UCL Institute for Women’s Health, University College London, Gower Street, London, WC1E 6BT UK; 13 Faculty of Medical and Health Sciences, University of Auckland, Private Bag 92019, Auckland, New Zealand; 14 Guy’s and St Thomas’ NHS Trust, Westminster Bridge Road, London, SE1 7EH UK; 15 West Middlesex University Hospital, Chelsea & Westminster Hospital NHS Foundation Trust, Twickenham Road, Isleworth, TW7 6AF UK; 16 Fetal Medicine Unit, St George’s University Hospitals NHS Foundation Trust, Blackshaw Road, London, SW17 0QT UK; 17 Molecular & Clinical Sciences Research Institute, St George’s University of London, Cranmer Terrace, London, SW17 0RE UK; 18 Reproduction and Perinatal Centre, Faculty of Medicine and Health, University of Sydney, Sydney, NSW 2145 Australia

**Keywords:** Implementation, Small-for-gestational age foetus, Antenatal screening, Process evaluation, Context, Acceptability, Feasibility, Cluster-controlled trial

## Abstract

**Background:**

Reducing the rate of stillbirth is an international priority. At least half of babies stillborn in high-income countries are small for gestational-age (SGA). The Growth Assessment Protocol (GAP), a complex antenatal intervention that aims to increase the rate of antenatal detection of SGA, was evaluated in the DESiGN type 2 hybrid effectiveness-implementation cluster randomised trial (*n* = 13 clusters). In this paper, we present the trial process evaluation.

**Methods:**

A mixed-methods process evaluation was conducted. Clinical leads and frontline healthcare professionals were interviewed to inform understanding of context (implementing and standard care sites) and GAP implementation (implementing sites). Thematic analysis of interview text used the context and implementation of complex interventions framework to understand acceptability, feasibility, and the impact of context. A review of implementing cluster clinical guidelines, training and maternity records was conducted to assess fidelity, dose and reach.

**Results:**

Interviews were conducted with 28 clinical leads and 27 frontline healthcare professionals across 11 sites. Staff at implementing sites generally found GAP to be acceptable but raised issues of feasibility, caused by conflicting demands on resource, and variable beliefs among clinical leaders regarding the intervention value. GAP was implemented with variable fidelity (concordance of local guidelines to GAP was high at two sites, moderate at two and low at one site), all sites achieved the target to train > 75% staff using face-to-face methods, but only one site trained > 75% staff using e-learning methods; a median of 84% (range 78–87%) of women were correctly risk stratified at the five implementing sites. Most sites achieved high scores for reach (median 94%, range 62–98% of women had a customised growth chart), but generally, low scores for dose (median 31%, range 8–53% of low-risk women and median 5%, range 0–17% of high-risk women) were monitored for SGA as recommended.

**Conclusions:**

Implementation of GAP was generally acceptable to staff but with issues of feasibility that are likely to have contributed to variation in implementation strength. Leadership and resourcing are fundamental to effective implementation of clinical service changes, even when such changes are well aligned to policy mandated service-change priorities.

**Trial registration:**

Primary registry and trial identifying number: ISRCTN 67698474. Registered 02/11/16. 10.1186/ISRCTN67698474.

**Supplementary Information:**

The online version contains supplementary material available at 10.1186/s13012-022-01228-1.

Contributions to the literature

- This is the first independent robust implementation study of GAP (Growth Assessment Protocol); we identified concerns about costs and staffing resources required for GAP implementation.
- Ambivalence about the value of GAP also appeared to impact upon staff willingness to implement, emphasising the need for consistently articulated leadership support.
- Our research shows how use of routine clinical data within a trial can identify gaps in implementation and inform future implementation research.
- Further methodological research is required on the development of composite measures of implementation strength.
- This is one of the first process evaluations to use the context and implementation of complex interventions framework.

## Background

Reducing stillbirth is an international priority [1, 2]. The stillbirth rate in the UK remains one of the highest in developed countries, despite reductions from 5.7/1000 in 2003 to 3.9/1000 in 2020 (England and Wales) [3, 4]. Up to 57% of stillbirths occur in foetuses who are small-for-gestational age (SGA, < 10th weight centile for gestational age) [5, 6], but less than half of SGA babies are detected antenatally (the rate varies by screening pathway but is between 21 and 50%) [7–14]. The English strategy to reduce stillbirth includes implementation of the national Saving Babies’ Lives care bundle with five components that target the following: the detection and management of SGA foetuses, maternal smoking cessation, early review for maternal concerns regarding reduced foetal movements, intrapartum foetal monitoring and preterm birth prevention [15]. These strategies are also common to other high-income countries [16].

The Growth Assessment Protocol (GAP), is a complex antenatal intervention developed and provided by the Perinatal Institute in Birmingham, UK. GAP aims to improve the rate of antenatal detection of the SGA foetus and thereby reduce the rate of stillbirth. In addition to strategies also set out by the Saving Babies’ Lives care bundle, GAP offers training materials, implementation support, guidance for stratification of pregnant women by risk of SGA and risk-appropriate surveillance of foetal growth (both of which are similar to strategies of the Saving Babies’ Lives care bundle), assessment of foetal growth according to customised standards (these use characteristics of the mother: height, weight, ethnicity and parity, and baby: sex, gestational age) and a standardised tool for auditing cases of missed SGA [17]. The DESiGN trial is the first and only randomised controlled trial comparing GAP to an alternative intervention. The clinical effectiveness trial found that GAP did not increase the rate of antenatal detection of SGA (the primary outcome), when compared to standard care [18]. This paper reports implementation outcome findings from the nested process evaluation and considers these in the light of the findings from the effectiveness study.

The Medical Research Council (MRC UK) guidance on evaluation of complex interventions such as GAP advises that process evaluation is key to understanding effectiveness in everyday practice [19]. Process evaluation can be used to assess fidelity of implementation, generate hypotheses on mechanisms of impact and identify contextual factors that are associated with different outcomes [20]. Evaluating implementation through hybrid-effectiveness trials is necessary to prevent type 3 error (the dismissal of an intervention because of failure to implement it as intended) [21]. The aims of the process evaluation in the DESiGN trial were to examine implementation outcomes, identify contextual factors and mechanisms of impact and understand how the intervention functions by describing it, its delivery and strategies used to implement it.

## Methods

The protocol for the DESiGN trial and manuscript reporting clinical effectiveness of GAP compared to standard care as seen in the trial have previously been published [18, 22]. This manuscript has been written according to the recommendations of the standards for reporting implementation studies (STaRi) statement [23], the full checklist is included in Additional file 1.

### Study design

The DESiGN trial utilised a hybrid type 2 effectiveness and implementation design [24, 25]. The primary aim was to examine the clinical effectiveness of the GAP intervention through the cluster RCT [18]. The secondary aim was to assess implementation outcomes, including implementation strength, through a mixed-methods process evaluation. We explored feasibility and contextual elements of implementation through a qualitative study and assessed implementation strength (incorporating the outcomes fidelity, dose and reach) using documentary analysis, training records and notes audit.

DESiGN was a pragmatic trial in which the intervention (which was already widely used in UK clinical practice without specific funding) was implemented as it would have been in the real world, without implementation support or funding from the trial team. Cluster sites in England, UK (11 of 13 based in London, a region in which uptake of GAP was low), were randomly allocated to implement GAP (seven clusters) or standard care (six clusters). Prior to contracting the GAP provider, two sites allocated to GAP implementation withdrew from the trial. These sites were excluded from the primary analyses of the trial. All women with singleton non-anomalous pregnancies during the trial period (starting from cluster randomisation between November 2016 and July 2017 and ending on 28 February 2019) were exposed to the intervention.

**Fig. 1:**
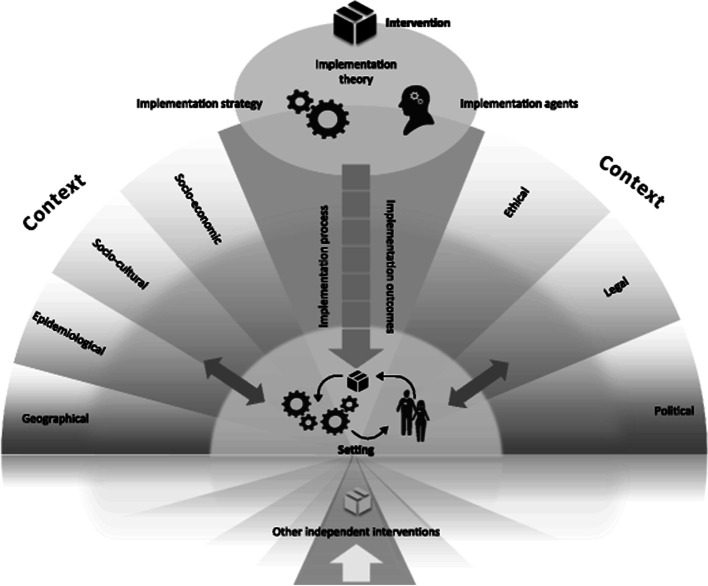
The context and implementation of complex interventions (CICI) framework. The framework comprises the three dimensions context, implementation and setting. The context comprises the seven domains: geographical, epidemiological, socio-cultural, socio-economic, ethical, legal, political context. Implementation consists of implementation theory, implementation process, implementation strategies, implementation agents and implementation outcomes. In the setting, the intervention and its implementation interact with the context. Reproduced under the terms of the Creative Commons Attribution 4.0 International License (http://creativecommons.org/licenses/by/4.0/), from Pfadenhauer et al (2017, Implementation Science) [29]

The implementation process evaluation drew on the Medical Research Council guidance for trials of complex interventions [19, 26] and was designed using the Consolidated Framework for Implementation Research (CFIR) evaluation framework [27]. Implementation outcomes were drawn from both Steckler and Linnan’s framework for process evaluation of public health interventions and research and Proctor et al.’s implementation outcome definitions (studied domains are detailed in Table 1 as applied to implementation of GAP) [27–29]. CFIR domains and constructs were then incorporated into interview schedules (see Table 1). The approach of measuring implementation strength (a term that encompasses implementation fidelity, dose and reach to present an overall indication of implementation) is a relatively novel interpretation of the literature, informed by a review by Schellenberg et al. (2012) [30]. The context and implementation of complex interventions (CICI) analytical framework was also used to provide additional granularity on context and implementation outcomes (Fig. 1). The CICI framework is designed to be used for process evaluation of complex interventions (such as GAP); it builds on and incorporates knowledge from previous frameworks, including CFIR, and provides an in-depth approach to assess context, using seven domains at three levels (micro, meso and macro) [29].

**Table 1:** Sources of data on GAP implementation outcomes at implementing sites

Implementation outcome	Outcome source	Application to implementation of GAP	Data source
Context±	Steckler and Linnan (2002) [28]; CFIR [27]; Pfadenhauer (2012) — for granularity of context [29]	Qualitative data collection instruments incorporated CFIR implementation domains and associated constructs [27]; framework analysis of macro, meso and micro context conducted using the CICI framework [29]	Semi-structured interviews with lead clinicians and frontline staff
Fidelity	Steckler and Linnan (2002) [28]	Adherence to GAP provider training requirement that 75% of staff from each professional group (midwives, sonographers, obstetricians) were trained using both (i) face-to-face and (ii) e-learning methods	Staff training records from the GAP provider
		Degree of concordance to Perinatal Institute guideline assessed as follows: *Low*: partial or no inclusion of Perinatal Institute’s (PI) recommendations throughout the guidelines, affecting over half of the recommendations. *Medium*: Moderately concordant with partial or no inclusion of PI’s recommendations in less than half of the recommendations *High*: Very concordant with only occasional differences where PI’s recommendations were partially included	Local clinical guidelines on screening for foetal growth anomalies
		Proportion of women correctly risk stratified (according to GAP)	Review of the maternity records of 600 women who gave birth during the trial period (40 from each of December 2018, January and February 2019 in each cluster)
Reach	Steckler and Linnan (2002) [28]	Proportion of women with a GAP-GROW chart in the notes	Maternity records review (see above)
Dose delivered and received	Steckler and Linnan (2002) [28]	Proportion of low-risk women* who had at least the minimum expected fundal height measurements performed and plotted on the chart	Maternity records review (see above)
		Proportion of low-risk women* referred for growth scan when indicated	
		Proportion of high-risk women* who had at least the minimum expected growth scans performed and plotted on the chart	
Implementation strength	Schellenberg et al. (2021) [30]	Combined assessment of fidelity, dose and reach	
Acceptability	Proctor et al. (2011) [31]	Acceptability of GAP implementation from the perspectives of clinicians	Semi-structured interviews with lead clinicians and frontline staff
Feasibility	Proctor et al. (2011) [31]	The degree to which GAP implementation is feasible, from the perspectives of interview participants	

*CICI* context and implementation of complex interventions framework, *GAP* Growth Assessment Protocol, *GROW* gestation-related optimal weight. *Risk status as determined by clinician. Risk assessment is expected to consider the risk stratification protocol specified in the GAP guidelines but may be modified for local practice. ±Assessed at both implementing and standard care sites

### Description of standard care

As described previously, clinical care in the standard care arm of the trial was not prespecified except that these clusters were expected to not implement GAP or use customised centiles for fundal height or foetal growth monitoring [18]. Clinical guidelines were collected from clusters allocated to continuation of standard care. A comparison between these and the GAP intervention is included in Additional file 2.

### Description of the intervention and implementation strategy

The intervention components and strategies for GAP implementation are summarised in Table 2. Further details have been summarised using the Template for Intervention Description and Replication (TIDiER) guidance in Additional file 3. A logic model describing the strategies by which GAP is expected to have an effect was conceived by the trial team and has been included in Additional file 4.

**Table 2:** Implementation process, intervention components and implementation strategies of the Growth Assessment Protocol, as specified by the intervention provider

Implementation process domains (CICI)	Intervention components	Implementation strategies
Decision to adopt		• Recruit sites
Planning and preparation	• Update the maternity unit’s foetal growth assessment guideline in line with guidance issued by the Perinatal Institute • Audit of baseline rates of detection of the SGA foetus • Trust protocol aligned with GAP	• Identify maternity unit’s GAP team and administration leads (midwife, sonographer, obstetric leads, information technology liaison for hardware and software) • Perinatal Institute convenes monthly meetings between nominated GAP leads from local sites to discuss implementation progress and challenges • Complete baseline audit of rate of SGA, referral for suspected SGA and confirmed SGA detection (3 months’ births)
Initial implementation	• Annual whole-staff training on the intervention by both face-to-face and e-learning methods	• Selected staff to attend GAP ‘train the trainers’ workshop, led by the Perinatal Institute • Trainers to cascade both face-to-face and e-learning GAP training to 75% of staff from each professional group: midwives, sonographers and obstetricians • Perinatal Institute continues to meet monthly with GAP leads
Full implementation (‘going live’)	• Risk stratification of pregnant women in early pregnancy into two strata according to whether women are at low or high risk of SGA, using the NHS-England risk-stratification decision tool [15] • Serial fundal height measurements for low-risk women, plotted onto a ‘gestation-related optimal weight’ (GROW) centile chart, which is customised by maternal height, weight, ethnicity and parity [32] • Serial foetal growth ultrasound for high-risk women, with the estimated foetal weight plotted onto the GROW chart • Protocols for the interpretation and onward management of plots on the GROW chart which deviate from the expected growth trajectory	• Use GAP SGA risk assessments and SGA management referrals from ‘go live’ date • Facilitate printing of GROW centile chart and incorporation into individual maternity notes • For low-risk women, begin plotting fundal height measurements onto GROW chart from 26 to 28 weeks every 2–3 weeks • For high-risk women, foetal growth ultrasound every 3 weeks from 26 to 28 weeks until the end of pregnancy • Raise awareness amongst staff of GAP with posters, emails, reminders and in-person visits by GAP leads and trainers to antenatal care settings • Liaise with PI about GAP queries
Evaluation, reflection and sustainment	• Guidance on the conduct of missed case audit and investigation	• Undertake audit of missed FGR cases (10 cases 6 monthly or 1% of birth rate)

### Data collection

To achieve the planned aims, both quantitative and qualitative data were required. The data sources are summarised in Table 1.

For the evaluation of implementation context, processes and intervention acceptability and feasibility, qualitative data were collected through semi-structured interviews conducted with a purposive sample of staff from each of the five implementing clusters; this included one clinical lead for GAP implementation from each professional group (obstetricians, midwives and sonographers) and a sample of frontline midwives and sonographers from each site (planned 40-50 interviews in total). A smaller sample of interviews (planned 6–12 interviews) was conducted with clinical leads at sites randomised to continue standard care. These interviews were designed to explore the extent to which standard care sites had implemented the five components of the Saving Babies’ Lives care bundle (NHS England) and to gauge service leads’ views about future implementation of GAP at the standard care sites.

The topic guides for the interviews are supplied in Additional file 5. Interviews and analyses were conducted before the results of the main trial were known. Interviews with all frontline staff were conducted by SR, an obstetric training-grade doctor. Interviews with all clinical leads were conducted by KC, an experienced qualitative researcher with a clinical background in midwifery. Where possible, interviews were conducted face to face or by phone if preferred. Interviews were recorded electronically and transcribed professionally. Transcript quality was checked and accordingly edited by the responsible interviewer. The transcribed, anonymised interviews were analysed using NVivo v11.0.

To assess strength of implementation, guidelines produced by site clinical leads for GAP implementation were collected from the sites, and staff training records were collected both from the sites and the Perinatal Institute. Local clinical guidelines for antenatal screening of foetal growth anomalies were also collected from sites randomly allocated to continue standard care. Remaining processes were assessed through a review of clinical notes for babies born during the trial comparison period. Forty women’s maternity records were randomly selected for each of 3 months (December 2018, January and February 2019) from the postnatal records stores at each implementing site. The sample size was chosen following a subjective assessment of the number of notes required to draw robust conclusions on implementation strength conducted by a senior researcher experienced in implementation science and a pragmatic decision regarding feasibility and staffing resource. Data were collected on women’s demographics and risk factors for SGA, clinician assessment of risk, the presence of a GROW chart, number of fundal heights measured and recorded (only counted if a minimum of 2 weeks apart and after 26 weeks’ gestation) and foetal growth scans plotted on the chart (minimum 3 weekly from 26 weeks) and evidence of a deviation in the foetal growth trajectory.

GAP guidelines do not provide definitions for slow and accelerative growth; these are assessed subjectively. To assess whether there was subjective evidence of a true deviation in the growth trajectory (as opposed to normal inter- or intraobserver variation), two senior obstetric training-grade doctors discussed the GROW charts for the first 80 cases and agree whether the plotted measurements featured a ‘possible’ (likely representing normal variation) or ‘definite’ (acute change) deviation from the expected foetal growth curve.

### Measurement of implementation strength

The methods for measuring each component of implementation strength are detailed in Table 1. The demographic data of women included in the notes review were summarised using number/percentage (*n*/%) for categorical data and median/interquartile range (IQR) or mean/standard deviation (SD) for continuously reported data. For each implementation outcome quantitatively assessed, the proportion of women meeting the expected criteria was reported using *n*/%. We later hypothesised that multiparous women were less likely to receive the expected number of fundal height measurements because a maximum 3-weekly fundal height measurement protocol does not fit with the current UK NICE schedule of antenatal care for these women [33]. For this measure, a post hoc comparison was made according to parity status using the chi-squared test.

We intended to build an overall score of implementation strength, to enable a sensitivity analysis of the clinical effectiveness of GAP. We were not able to find evidence within the literature, nor after consulting experts in this field, on the relative weight of each element of implementation strength to apply in the scoring system. We therefore determined to present the scores for each measure individually only.

### Qualitative implementation data analysis

Interview data were deductively coded by two independent researchers (SR and KC) using the context, implementation and setting dimensions of the CICI framework. The analysts regularly discussed and documented coding decisions using NVivo ‘memos’, to enhance procedural rigour and inter-researcher consistency (see Additional file 6) [34]. Where the data did not fit clearly into the available codes, the two analysts discussed with a senior qualitative researcher (JS) and, if required, added subcodes within existing CICI domains. For example, we added a ‘feasibility’ code within the implementation outcome domain of ‘acceptability’. During the analysis, we were guided by Proctor and colleagues’ definitions of acceptability and feasibility [31]. When the main analysis was complete, both analysts conducted a further analysis of the database, to explore interactions between context domains and GAP implementation processes. We employed a priori thematic saturation (as described by Saunders et al.) [35] and judged this to have occurred where detailed, in-depth data from a range of different participants and sites provided confirmation of the CICI framework domains.

### Ethical considerations

Ethical approval for this trial was obtained through the Health Research Authority Integrated Research Applications System from the London Bloomsbury Research Ethics Committee (Ref. 15/LO/1632) and the Confidentiality Advisory Group (Ref. 15/CAG/0195).

Since the numbers of sites implementing GAP (*n* = 5), withdrawing early prior to GAP implementation (*n* = 2) or continuing standard care (*n* = 6) were small, there is a risk that sites or participants could be identifiable in this paper. Both sites and participants are referred to with pseudonyms. Wherever possible, key site characteristics have been omitted, and staff are referred to either as ‘frontline workers’ (midwives, sonographers) or ‘GAP leads’ (clinical specialists involved in organisational leadership and executive decision-making) to minimise the likelihood of recognition.

## Results

In total, 55 qualitative interviews were conducted including 27 interviews with clinical leads (22 GAP leads at implementing sites and 5 service leads at standard care sites) and 28 interviews with frontline staff (implementing sites only). The interviews took place between February 2018 and May 2019. All interviews were conducted following initial implementation at cluster sites. Three interviews were conducted by phone. We were unable to arrange interviews with either lead or frontline sonographers at one implementing site, but interviews were conducted with all the intended professional groups at all other sites.

### Findings

Firstly, we report findings from our qualitative inquiry on two key implementation outcomes, ‘acceptability’ and ‘feasibility’, and outline the influence of context on GAP implementation during the DESiGN trial (see Additional files 7 and 8 for more extensive qualitative data). Verbatim quotes are included, but text presented within square brackets has been summarised for brevity or clarity. These qualitative data provide context for the quantitative data on measures of implementation strength, which we then present.

### Acceptability: GAP lead and frontline staff perspectives on the potential value and clinical effectiveness of GAP

Within the CICI framework, staff are considered ‘individual implementation agents’ by virtue of being ‘actively involved in…administering or implementing an intervention’ [29]. Staff perspectives about the value and effectiveness of GAP were important throughout the implementation process. We found evidence of differing views amongst frontline staff and GAP leads about whether GAP was beneficial for women or likely to increase detection of SGA. Some felt the GAP approach was promising:

I generally welcomed it, I was excited about it, I thought it was…a nice rigorous way of decision-making.

(SC21, GAP Lead, Site 10)

Others were more negative:

Generally, I think the majority of us don’t really want to [implement GAP]. We don’t really understand why we are doing it.…

(HP12, Frontline staff, Site 11)

I didn't welcome it. I was a bit sceptical of it and maybe this was influenced from speaking to some of my colleagues….

(SC31, GAP Lead, Site 9).

GAP leads had often attended ‘train the trainer’ events held by the Perinatal Institute months before implementation began. They also learned about GAP through clinical networks, conferences and publications. Frontline staff usually learned about GAP through face-to-face or online training provided by GAP leads. However they felt about GAP, respondents usually agreed it was important to do the DESiGN trial to address this question (see Additional file 7).

### Acceptability of GAP implementation: GAP lead and frontline staff perspectives

Frontline staff felt that GAP was a useful intervention because plotting foetal growth onto a customised chart was straightforward, and they hypothesised that this would improve detection of SGA, reduce variations in care by standardising practice and possibly reduce routine interventions. Staff felt that customised charts were acceptable to the women they cared for, and that both the work-based ‘face-to-face’ and online (Perinatal Institute e-learning package) trainings were good. However, providing GAP training was problematic for organisations (see ‘Feasibility of GAP implementation: staff perspectives’).

Whilst some GAP leads believed GAP enhanced standardised assessment of foetal growth, others expressed concerns about a variety of issues, including plotting errors (see Additional file 7). A key issue for both frontline staff and leads was that GAP identified potentially large babies without a corresponding care pathway. Staff felt this could increase women’s anxiety and potentially lead to increased interventions.

[GAP leads to identification of more large babies and therefore] lots of intervention that may not be warranted.

(HP3, Frontline staff, Site 7)

I’d say probably half of the women are coming above the line... I just think a lot of women come up quite high on the chart and it can be quite worrying for them

(HP91, Frontline staff, Site 10)

Leads noted that GAP could also create clinical confusion. Examples included a new focus on estimated foetal weight over foetal abdominal circumference, as sonographers had been taught, and there were differences noticed between what was taught in GAP training and recommendations in implementing site protocols (see ‘assessment of fidelity’).

The sonographers were very uncomfortable with not allowing the AC [fetal abdominal circumference] to drive the decision around further scanning….…[they]…felt that they might get blamed if, you know, the EFW [estimated fetal weight] is normal but the AC is slightly dropping and they didn’t act accordingly

(SC17, GAP lead, Site 11)

Overall, frontline staff found the GAP intervention to be acceptable, despite some reservations, and GAP leads agreed there were benefits, but GAP leads were also aware that GAP introduced new clinical complexity. Frontline staff cited access to good quality training, ease of implementation, and benefit to women, all of which contributed to a sense that GAP was potentially a useful approach to improve SGA detection. Some aspects were considered less satisfactory by frontline staff and GAP leads; these included identification of larger babies, measurement errors, clinical uncertainty, and potential to increase interventions. The ‘acceptability’ data are drawn from 43 interviews and 180 extracts, reflecting good saturation of domain codes, with consistency across sites and participant groups (see Additional file 7).

### Feasibility of GAP implementation: GAP lead and frontline staff perspectives

Whilst clinical staff generally considered GAP to be acceptable and beneficial, the feasibility of actually implementing GAP often appeared conditional. For example, training was feasible *if* there were sufficient staff to provide cover; GAP could be implemented *if* a dedicated ‘champion’ could focus on this. Key feasibility concerns identified by frontline staff and GAP leads included the anticipated and observed increase in ultrasound scans required, which impacted on sonographer breaks, clinics running late, and led to ‘breaches’ of other clinical targets:

We have a lot of patients who come and usually we are full, we are booked completely, and to fit the patient within three working days is very, very difficult. Sometimes we have to scan during our lunchtime which is not ideal at all but then otherwise we breach the time…

(HP41, Frontline staff, Site 9)

GAP leads also reported how shortages of scan slots and sonographers led to decisions with lower concordance between local and GAP guidelines:

Er, their [GAP] BMI [body mass index, referral point] is, er, lower than ours, so we would only refer if they were 35 and over. Just because all of our women…we’d just be referring everyone

(SC20, GAP lead, Site 7)

Frontline staff also reported that using GAP sometimes meant appointments took longer, due to plotting time, having to hunt for charts or missing information or to seek additional advice or a second opinion, and sometimes this meant that clinics over-ran. Frontline staff and leads also reported problems with accessing information technology (IT, lack of printers or computers in hospital or community settings) or equipment.

During implementation, GAP leads were concerned about the feasibility of providing face-to-face training or releasing staff to undergo online training.

…it’s just not feasible for myself and my colleague to train [hundreds of] midwives between the two of us, when we’re not being given any allocation of time…

(SC06, GAP lead, Site 9)

We have had drop-ins where we get people to try and sit and do their online training. And I think that has been the biggest issue, as far as I know we’re still not at the level that we should’ve… had with the online training.

(SC21, GAP lead, Site 10)

Frontline clinicians and GAP leads also reported software duplication and non-alignment between GAP training and site protocols, practice or software:

So we are using [ultrasound generated charts] in conjunction with the GAP charts still…at the moment, they are running alongside each other which at the beginning did generate some problems…

(HP23, Frontline staff, Site 11)

…the [Trust] IT system doesn’t link in with the Perinatal Institute’s GAP GROW, which is possibly the case for a lot of people’s IT systems…So you end up with lots of bits of paper [laughs] because it’s a bit of a hybrid, and probably every trust has to work out their own little system for that.

(SC04/SC07, GAP lead, Site 8)

Despite these feasibility concerns, GAP leads and frontline staff were committed to improving detection of SGA and worked hard to find solutions to the issues they had identified; for example providers increased scanning capacity ready to introduce GAP, leads planned ahead and wrote business cases for additional sonography staff and resources and sought to give staff protected time to do e-learning (see Additional file 7).

### Describing the context of implementation and how it interacts with the implementation process

We conducted a further analysis to examine implementation as a chronological process and document the impact of context; we separated this data into micro, meso and macro levels, using the definitions provided in the CICI framework (see Additional file 8) [29]. Contextual factors affected the early stages of implementation (‘exploration and decision to adopt’ and ‘planning/initial implementation’) and continued to impact during ‘full implementation’. After ‘full implementation’, there were no new observations about the influence of context, perhaps because most interview questions were focused on the implementation phases, but the concerns identified appeared likely to have an impact on the longer-term sustainment of GAP within implementing organisations.

### How context affected early implementation

During the planning stage, the external ‘macro’ context appeared influential; GAP leads are regularly referred to targeted national campaigns and policies designed to raise awareness about the UK’s relatively high stillbirth rates [15, 36, 37].

There were multiple triggers, some of them being our own local experiences in reviewing cases where there had been adverse outcomes…That was one trigger. Then the growth assessment guidelines from [RCOG]…was another trigger. Then the Saving Babies’ Lives processes also needed us to look at ways of streamlining our care. Those are the kind of things I would say made us choose [to adopt GAP].

(SC12 and SC22, GAP lead, Site 8)

Lead clinicians at ‘standard care’ sites also identified the national policy context as influential. Post-randomisation, clinicians at these sites also needed to identify ways to respond to the same policy guidance without implementing GAP or using customised charts (Additional file 8).

Whilst there was a national consensus that current practice to detect SGA and prevent stillbirth was problematic, clinicians differed in their views of whether it was possible to implement GAP without additional resources. Two provider organisations were randomised to implement GAP but did not implement the intervention, and financial considerations appeared to have influenced these decisions:

…our Trust is under pressure with finances, so they are cutting down everything. So, that is why the new management didn’t want to spend this additional [money] for the GAP programme. It’s not my decision, it’s a management decision.

(SC1, Clinical lead, Site 13)

…the Research & Development department did try their best, but then when they saw there was no funding, they couldn’t see any value in [participating in the trial]…[but] we see the benefit, the benefit of the trial.

(SC14, Clinical lead, Site 12)

During the early implementation stage (see Table 2), interactions between context and implementation occurred mainly at the ‘meso’ (organisational) level (see Additional file 8). Organisations experienced delays and barriers, mainly due to staff shortages, pressures of work and problems identifying GAP leads with sufficient capacity to invest the time needed. Interviewees also identified strategies and contextual factors that had helped with implementation, such as supportive relationships with colleagues and interdisciplinary working, which meant that staff helped each other to understand and implement new protocols.

Int: Did you find that there was anything that made it easier for you, or something that was supporting you to cascade training to your colleagues?

I think the support that we got from [colleague 1] and also [colleague 2] was very, very helpful. And [colleague 1] was very visible to us and …very willing to answer a question…

(HP5, Site 11)

### Impact of context on ‘full implementation’

Context had a notable impact on full implementation (see Table 2), mainly at either the ‘micro’ (individual) and ‘meso’ levels (see Additional file 8). At the ‘micro’ level, it became clear how the acceptability issues identified by staff, and discussed earlier, might impede implementation. The impact of changes to usual practice brought by GAP could be seen, as clinicians began to experience dissonance between what they would previously have done and what they should ‘now’ do, according to GAP:

…our protocol has been historically –[for] 50 years, ever since ultrasound assessment has been [used], practice has been [to scan again in] four weeks, so bringing it down to three weeks …is a bit hard

(SC22, GAP lead, Site 8)

…you know, [at] 36 weeks, and you measure 33 centimetres, your mind tells you, I have to scan this woman! [laughs] But the chart tells you, you don’t need to. So for the midwives it’s a bit of …you know, they have to really feel confident that actually yes, it’s working

(SC25, GAP Lead, Site 10)

On the other hand, staff were also motivated to implement GAP to improve care:

…there were babies being missed [before] and the outcomes were not good for those babies, so [GAP] definitely needed to be implemented.

(HP74, Frontline staff, Site 7)

Context could either impede or favour implementation (see Additional file 8); the micro-contextual analysis showed how staff reflected individually on ‘missed’ SGA cases, and this meant they were receptive to an intervention which might improve care. The work undertaken by staff in response to the organisational (‘meso’) context particularly demonstrated the additional time and workload implications as managers and senior leads attempted to resolve day-to-day implementation obstacles. There were also examples where executive boards or directors had approved extra funding for staff or capital expenditure, allowing implementation to proceed, but staff felt these only partially addressed the shortfalls identified.

### Measure of implementation strength

Implementation strength was measured using guidelines on screening for SGA which were collected from all five implementing sites, training records received from the GAP provider and a review of 595 maternity records for babies born during December 2018–February 2019. The demographic characteristics of the women whose maternity records were reviewed are summarised in Additional file 9.

#### Fidelity

All five sites achieved the target of training > 75% staff members from each professional group in face-to-face methods on the GAP intervention; but only one site achieved the e-learning target (Table 3). This may be explained by the acceptability and feasibility findings on training, whereby some members of staff felt that the e-learning training was unnecessary, and GAP leads found it difficult to release staff from clinical duties for additional training.

**Table 3:** Overall assessment of implementation strength

			Site 7	Site 8	Site 9	Site 10	Site 11
**Fidelity**	Degree of concordance^a^ with Perinatal Institute guideline		Low	High	Medium	Medium	High
	Proportion of staff trained within each professional group	Face-to-face target	> 75%	> 75%	> 75%	> 75%	> 75%
		E-learning target	< 75%	< 75%	> 75%	< 75%	< 75%
	Proportion of women risk stratified according to GAP		87.5% (105/120)	78.6% (92/117)	84.2% (105/121)	83.2% (99/119)	84.4% (98/116)
**Reach**	Proportion of women with a GAP-GROW chart in the notes		62.2% (74/119)	98.3% (115/117)	93.3% (131/121)	96.6% (115/119)	94.2% (113/120)
**Dose**	Proportion of low-risk women who had at least the minimum expected number of fundal height measurements performed and plotted on GROW		8.2% (4/49)	53.2% (42/79)	34.4% (31/90)	31.4% (22/70)	18.1% (15/83)
	Proportion of low-risk women referred for growth scan when definite plot deviation		40.0% (4/10)	79.2% (19/24)	80.9% (17/21)	66.7% (10/15)	61.2% (19/31)
	Proportion of high-risk women who had at least the minimum expected number of growth scans performed and plotted on GROW		0.0% (0/33)	16.7% (8/48)	2.9% (1/35)	12.8% (6/47)	5.3% (2/38)

^a^Degrees of concordance defined in Table 1

The assessment of concordance with GAP guidelines identified wide variation. The guidelines from two of the five implementing sites were assessed as having high fidelity to the recommended GAP guideline; one site guideline had low fidelity (Table 3). Low or medium fidelity was usually caused by adaptations made to the local guidance, prioritising women with some risk factors over others and reducing the frequency of (or removing the recommendation entirely for) ultrasound scans offered to those women. This finding may be partly explained by the qualitative data on acceptability and feasibility of implementing the intervention, in particular the finding that maternity services were concerned about a shortage of ultrasound appointments and sonographers. A detailed breakdown of the deviations from the GAP recommended statements is available in Additional file 10.

Maternal risk of SGA, assessed as per GAP guidance, was compared to that allocated by the assessing clinician for agreement. There was agreement achieved in 84.9% (*n* = 505) cases. Of those women in whom there was disagreement, 19 women (21.1% of those with disagreement by GAP guidelines) had appropriate risk stratification according to local protocols. Results by risk status and by site are detailed in Table 4.

**Table 4:** Outcome of the assessment of risk stratification, comparing clinician assessment to GAP and local recommendations

		Site reference					
	Risk status (by GAP)	Site 7	Site 8	Site 9	Site 10	Site 11	All
Agreement between GAP and clinician	High risk (*n*)	32	24	21	32	24	133
	Low risk (*n*)	73	68	87	68	76	372
	Both *n*(%)	105/120 (87.5%)	92/117 (78.6%)	108/121 (89.3%)	100/117 (85.5%)	100/120 (83.3%)	505/595 (84.9%)
Clinician did not classify risk as recommended in GAP	High risk (*n*)	9	13	9	3	14	48
	Low risk (*n*)	6	12	4	14	6	42
	Both *n*(%)	15/120(12.5%)	25/117 (21.4%)	13/121 (10.7%)	17/117 (14.5%)	20/120 (16.7%)	90/595 (15.1%)
If GAP classification is wrong, classified correctly as per local policy?	*n*(%)	2/15 (13.3%)	0/25 (0.0%)	7/13 (53.8%)	7/17 (41.2%)	3/20 (15.0%)	19/90 (21.1%)

#### Reach and dose

With regard to the measures of dose and reach assessed by notes review, the proportion of women in whom the target was achieved is presented for each site in Table 3. For these measures, site 7 was consistently the lowest scoring site, and site 8 was the highest scoring for three of the four measures. There was evidence of a difference in the dose received between nulliparous and multiparous women (38.1% vs 21.9%, *p* < 0.001, Table 5) across all 5 sites implementing GAP. Overall, implementation reach was generally good, but the rate of dose delivered was low. The low dose delivered may be partly explained by the low or medium strength of fidelity, particularly when this relates to the offer or frequency of foetal growth scans for women at higher risk of SGA.

**Table 5:** Proportion of low-risk women with at least the minimum expected number of fundal height plots on GROW chart

	Women with at least the minimum expected number of fundal height chart plots	
Site identifier	Number	Percentage
Site 7 (*n* = 49)	4	8.2%
Nulliparous (*n* = 25)	3	12.0%
Multiparous (*n* = 24)	1	4.2%
Site 8 (*n* = 79)	42	53.2%
Nulliparous (*n* = 43)	28	65.1%
Multiparous (*n* = 36)	14	38.9%
Site 9 (*n* = 90)	31	34.4%
Nulliparous (*n* = 55)	22	40.0%
Multiparous (*n* = 35)	9	25.7%
Site 10 (*n* = 70)	22	31.4%
Nulliparous (*n* = 43)	15	34.9%
Multiparous (*n* = 27)	7	25.9%
Site 11 (*n* = 83)	15	18.1%
Nulliparous (*n* = 36)	9	25.0%
Multiparous (*n* = 47)	6	12.8%
Total (*n* = 371)	**114**	**30.7%**
Nulliparous (*n* = 202)	**77**	**38.1%**^**a**^
Multiparous (*n* = 169)	**37**	**21.9%**^**a**^

^a^Chi-squared test comparing proportion of nulliparous to multiparous women with the expected number of fundal height plots, *p* < 0.001

#### Overall

There was wide variation in the scores for each component achieved by sites. Site 7 consistently scored lowest for the majority of measures. There is no individual site which consistently scored highest for each implementation outcome; however, site 8 scored highest for the most implementation strength components.

## Discussion

### Summary of findings

The process evaluation identified that GAP was, in principle, acceptable to frontline staff and GAP leads, despite some negativity about the value of the intervention, but did not always prove feasible in the context of practice. The organisational context, including open articulation of both positive and negative views about GAP by individuals in lead roles, may have affected frontline staff confidence in GAP. This could have diminished willingness to implement GAP, even though staff had strong commitment to improving care quality and increasing SGA detection. Frontline staff and GAP leads also acknowledged that the DESiGN RCT was valuable in the context of clinical equipoise but were concerned about whether GAP was an appropriate use of scarce resources and recognised that to fully implement GAP, additional resource was required. Whilst macro-political context affected early decisions to adopt GAP, as implementation progressed, the impact of context was more evident at the organisational and individual levels.

With regard to implementation strength, we identified variation amongst the five cluster sites, although with greater variation for components of fidelity and dose than for reach. Overall, scores for implementation fidelity were variable by component measure; all sites achieved the face-to-face training target, and 78–87% of women were correctly risk stratified, but only one site met the e-learning target, and local guidelines were variably concordant. Most sites achieved high scores for implementation reach (median 84% of women had a GROW chart) but generally low scores for dose (median 31% of low-risk women and 5% of high-risk women were monitored for SGA as recommended, although a median of 67% of low-risk women were appropriately referred for a foetal growth scan when indicated). Dose was low amongst women at high risk of SGA because only one site protocol recommended 3-weekly scans between 28 weeks and birth; all other sites recommended 4-weekly or less often scans, and this has affected the fidelity measure. Both GAP leads and frontline clinical staff cited resource availability as a common cause of lower concordance with recommended practice.

### Interpretation and comparison with available literature

This process evaluation conducted as part of the DESiGN trial is the first published report of a study of GAP implementation which adopted standard implementation outcomes, as recommended by established implementation science guidelines. We observed that GAP implementation was acceptable to clinical staff but also hampered by feasibility issues, including resource constraints. Additional clinical time was needed for training, and staff believed that both GAP and the Saving Babies’ Lives care bundle recommendations for management of reduced foetal movement were together contributing to increasing demand for scans and to rising interventions (including earlier induction of labour). A longitudinal evaluation of the same care bundle suggested that organisations which implemented the care bundle reported an increase in the use of ultrasound scans (by 24%) and induction of labour (19.5%) [38].

A retrospective observational study conducted by Gardosi et al. (2020) categorised all UK maternity units into three groups — non-implementers, partial implementers and complete implementers — according to whether they were registered as GAP users and, if so, whether they consistently recorded birthweight centiles and pregnancy outcomes on GAP software for at least 75% of all births (complete implementers recorded 75% or over, partial implementers recorded rates lower than this threshold) [39]. Complete implementation was associated with a reduction in stillbirth when compared to non-implementation of GAP (3.99/1000 vs 4.37/1000, *p* = 0.04), whereas there was no difference in the rate of stillbirth between partial and non-implementers. In another study, clinical guidelines from all 15 non-DESiGN trial GAP implementing sites were found to be non- or partially compliant with four out of five components of the SGA detection element of the SBL care bundle. Inclusion of these components in local guidelines is also expected as part of GAP implementation, demonstrating that partial concordance with GAP guidelines is widespread in UK maternity units [40]. Our process evaluation was more detailed than either of these single assessments of implementation, by documenting the extent to which the implementing cluster sites complied with each element of the GAP intervention.

Our analyses were informed by the CICI framework, which encourages consideration of micro, meso and macro levels of context [29]. Whilst we had anticipated that wider national initiatives, such as the Saving Babies’ Lives care bundle, would impact on implementation of GAP, it was interesting to see how this played out within sociocultural ‘micro/meso’ context of implementing organisations. Our qualitative data demonstrated that staff were not always persuaded that the intervention was valued and supported by their peers and clinical leaders; hearing of others’ negativity towards GAP meant that some staff felt less sure that the initiative was ‘really’ useful, despite a uniform desire to improve detection of SGA and prevent stillbirth. This in turn engendered a sense of ethical or moral uncertainty, even dissonance, as staff described thoughts about whether GAP was the ‘right’ thing to do or whether there may be potential for causing unintentional harm or misappropriation of scant resources. In their evaluation of a large-scale organisational patient safety initiative, Benning et al. similarly observed that staff needed to believe that a proposed change would be an effective way to tackle the problem identified, and that visible leadership commitment to a new approach was important for implementation to succeed [41]. Similarly, in their process evaluation of a pragmatic cluster RCT within GP practices, McMullen et al. reported that both resource provision (in terms of staff and time) and clear and continued endorsement of the intervention by the clinical leadership are required for effective implementation [42]. This is consistent with Dixon-Woods’ observation that high-quality management and leadership are important to successful implementation, and that improvement ‘without the right contextual support is likely to have limited impact’ [43].

Implementation of GAP achieved in the DESiGN trial did not result in an increase in the rate of SGA detection, when compared to standard care [18]. We do not know whether the strength of implementation in GAP sites explains the DESiGN trial finding or whether the lack of effect is fully or partly explained by the intervention not being superior to standard care. Furthermore, we do not know whether each of the components or strategies of the complex GAP intervention are of equal importance in achieving improved detection of SGA.

The study of implementation strength is relatively novel in hybrid implementation-effectiveness trials, particularly whilst adopting mixed quantitative and qualitative methods. Schellenberg et al. (2012) identified that there was no consensus on how best to measure implementation strength, nor how best to present an overall assessment of strength [30]. Hargreaves et al. (2016) also expressed caution about the application of arbitrarily determined weights to component measures of implementation strength [44]. Furthermore, as noted in the MRC framework on process evaluation for complex interventions, such data integration is expected to be challenging, with significant limitations of statistical power, where assessment of implementation strength is based upon measures collected at only a few sites [45]. Similarly, because GAP is a complex intervention, we were unable to conclude which elements of the implementation strength measure might contribute with most weight to the overall effectiveness or to separate these from the effects of national policy implemented contemporaneously.

### Strengths and limitations

The strengths of the process evaluation reported for this trial lie in the comprehensive and mixed-methods assessment of a wide range of implementation outcomes. Through an innovative development of novel methodology, including case note reviews to investigate implementation strength, we have developed hypotheses to explain the non-superiority of GAP over standard care in the DESiGN trial. Strengths of the qualitative process evaluation included good recruitment that overall leads to collection of rich and detailed data.

This process evaluation was limited by the lack of guidance or literature on summarising implementation strength into a composite score and the low number of sites included in the cluster randomisation, preventing conduct of a sensitivity analysis to examine the relationship between the site-specific composite or outcome-level implementation strength and the clinical effectiveness of the GAP intervention. The assessment of fundal height growth trajectories that should trigger a foetal growth ultrasound scan was subjective, but this is also the case when using the intervention in routine practice. A study of the degree to which expected standard care was applied in practice was not undertaken, because it was not central to our aims in assessing intervention implementation outcomes but may have been useful to determine the extent to which challenges identified were more widespread. We were also limited by an inability to distinguish between the effects of the studied intervention and those of a national policy (SBL care bundle) with similar aims which was implemented simultaneously, including in clusters allocated to standard care. Similarly, whilst GAP had not previously been implemented in the study sites, it had been adopted in most maternity units outside London. We were unable to control for exposure of healthcare workers from standard care sites to GAP training and application if they had previously worked in one such maternity unit; nevertheless, they were expected to follow local guidelines and did not have access to GAP resources whilst working in standard care sites.

The qualitative inquiry was limited by difficulty in recruiting sonographers at the implementing site with lowest overall implementation strength and at those sites randomised to implement GAP but which did not implement, so we lack data on sonographer perspectives. Frontline obstetricians were not targeted for recruitment, except for where they acted as GAP leads, and so the staff perspectives are drawn more from sonographers or midwives providing routine care. We did not achieve recruitment of women, despite attempting this.

### Implications of the findings

Whilst the GAP intervention was found to be acceptable to most members of staff interviewed, its implementation was limited by a lack of adequate resource and by the perceptions of staff that their leadership teams were not completely behind the intervention. Whilst GAP was being implemented in the DESiGN trial with the intention of providing evidence from a randomised control trial on its effectiveness, it had already been widely implemented in the UK, and therefore, clinical leaders were aware of conflicting evidence for, and against, its implementation. We noted that implementation started long before any changes to practice were made, as staff absorbed information about ‘the problem’ and ‘the evidence’ and observed how their organisation’s leadership engaged with change. Our findings suggest that GAP leads were not all consistently supportive of implementation, partly due to concerns about staffing and resource but also because there was a lack of high-quality research evidence underpinning the GAP intervention, such as that from randomised controlled trials or meta-analyses. This illustrated the difficulties of gaining leadership support for implementation where evidence is lacking, and we suggest that these early stages require fuller consideration to enhance the likelihood of successful implementation. Our findings also demonstrate the importance of ensuring that implementation of a new intervention is adequately resourced, to ensure that it is feasible to implement it as intended.

Methodologically, our findings point to the need for evaluation techniques that could distinguish between the impact of separate and different improvement initiatives taking place simultaneously within organisations, including the separate components of complex interventions. This has previously been identified as a problem in other trial-based process evaluation [46]. Such interventions are not uncommon within healthcare, yet identifying the discrete impact of GAP elements within the context of the national SBL care bundle was problematic.

The implementation strength data were invaluable for interpreting trial outcomes and represent a methodological development towards assessing implementation strength which has potential to enhance the value of future process evaluation studies. Such studies could build on this method and undertake detailed prospective examinations of discrete measures of fidelity, reach and dose. The notes review conducted as part of the assessment of implementation strength was robust and integral to the overall conclusions drawn in this process evaluation. We recommend that researchers planning future hybrid type 2 trials also plan to assess the extent to which policies were actually implemented, rather than assessing this from self-reported data, both in implementation and standard care sites. This would provide invaluable data on whether challenges seen in intervention implementation were unique to the intervention or also seen more widely and the extent to which routine care in ‘standard care’ sites resembles the intervention being tested in the trial. Such assessments are time-consuming, and so research staff costs should be included in the study budgets for such trials. Evidence that lack of resources (time, staff availability, clinic slots) impacted on implementation strength also support that sufficient resources are needed to maintain intervention fidelity, reach and dose.

For future trials intending to conduct detailed assessments and draw conclusions regarding the elements of implementation strategy which result in improvements in clinical effectiveness, a large number of clusters are necessary. Methodological guidance is required on the assessment of implementation strength, including on sample sizes needed to determine how different elements contribute to overall effectiveness. This has also been identified as a problem in other cluster trial-based process evaluation [47].

## Conclusion

The Growth Assessment Protocol, a complex intervention implemented during the DESiGN trial, was found to be acceptable amongst staff, but with issues of feasibility caused by conflicting pressures on staff time, availability of resource and variable beliefs among clinical leaders with regard to the value of the intervention. These issues are likely to have impacted on strength of implementation, including the difficulties faced in achieving the e-learning targets and the variable concordance of local protocols to those recommended by the provider, with the resultant variation in dose of exposure received by women. However, women were generally risk assessed as recommended, and a high proportion of maternity records reviewed at all sites contained a GROW chart.

The importance of adequately resourcing changes to practice which are being introduced in the context of RCTs (including provision of results from cost-effectiveness evaluations that support allocation of resource to the intervention), and for consistently articulated leadership support commencing early in the implementation process, both found in previous implementation studies, are also borne out in this research. Further methodological development is needed to build on the novel and detailed measurement of implementation strength undertaken in this study; longer-term research might also consider measurement of implementation sustainment and identify elements of complex interventions that prove crucial for scale-up and wider adoption of novel care processes.

## Supplementary Information

**Additional file 1.** STARI checklist.

**Additional file 2.** Description of practice in clusters allocated to standard care.

**Additional file 3.** TIDIER description of the intervention.

**Additional file 4.** Logic model.

**Additional file 5.** Topic guides for interviews with clinical staff.

**Additional file 6.** Detail of approach to rigour used in DESiGN process evaluation and qualitative data analysis.

**Additional file 7.** Supporting data on acceptability and feasibility.

**Additional file 8.** Supporting data on context.

**Additional file 9.** Characteristics of women included in the notes review.

**Additional file 10.** Deviations of local guidelines from GAP recommendations.

## Data Availability

The datasets generated and/or analysed during the current study are not publicly available due the risk of identifying participants, but extracts are available from the corresponding author on reasonable request.

## Abbreviations

AC: Abdominal circumference (foetal)
CICI: Context and implementation of complex interventions (framework)
DESiGN: DEtection of the Small for GestatioNal age fetus
EFW: Estimated foetal weight
GAP: Growth Assessment Protocol
GROW: Gestation-related optimal weight (customised standard foetal and neonatal weight chart)
IQR: Interquartile range
IT: Information technology
PI: Perinatal institute (intervention provider)
SD: Standard deviation
SGA: Small for gestational age
STaRI: STandards for Reporting Implementation studies
TIDieR: Template for Intervention Description and Replication

## References

[1] Safer Maternity Care [https://www.gov.uk/government/publications/safer-maternity-care]. Accessed 4 Aug 2021.

[2] Lawn JE, Blencowe H, Waiswa P, Amouzou A, Mathers C, Hogan D, Flenady V, Froen JF, Qureshi ZU, Calderwood C (2016). Stillbirths: rates, risk factors, and acceleration towards 2030. Lancet.

[3] Characteristics of birth 1. England & Wales, 2013 [http://www.ons.gov.uk/ons/rel/vsob1/characteristics-of-birth-1%2D%2Dengland-and-wales/2013/index.html]. Accessed 28 Jan 2017.

[4] Provisional births in England and Wales: 2020 [https://www.ons.gov.uk/peoplepopulationandcommunity/birthsdeathsandmarriages/livebirths/articles/provisionalbirthsinenglandandwales/latest]. Accessed 4 Dec 2020.

[5] Small for gestational age fetus: investigation & management. Green-top Guideline No. 31 [https://www.rcog.org.uk/guidance/browse-all-guidance/green-top-guidelines/small-for-gestational-age-fetus-investigation-and-management-green-top-guideline-no-31/] 10.1111/1471-0528.17814 38740546

[6] Gardosi J, Kady SM, McGeown P, Francis A, Tonks A (2005). Classification of stillbirth by relevant condition at death (ReCoDe): population based cohort study. BMJ.

[7] Backe B, Nakling J (1993). Effectiveness of antenatal care: a population based study. Br J Obstet Gynaecol.

[8] Monier I, Blondel B, Ego A, Kaminiski M, Goffinet F, Zeitlin J (2015). Poor effectiveness of antenatal detection of fetal growth restriction and consequences for obstetric management and neonatal outcomes: a French national study. BJOG.

[9] Jahn A, Razum O, Berle P (1998). Routine screening for intrauterine growth retardation in Germany: low sensitivity and questionable benefit for diagnosed cases. Acta Obstet Gynecol Scand.

[10] Mattioli KP, Sanderson M, Chauhan SP (2010). Inadequate identification of small-for-gestational-age fetuses at an urban teaching hospital. Int J Gynaecol Obstet.

[11] Kean L, Liu D (1996). Antenatal care as a screening tool for the detection of small for gestational age babies in the low risk population. J Obstet Gynaecol.

[12] Chauhan SP, Beydoun H, Chang E, Sandlin AT, Dahlke JD, Igwe E, Magann EF, Anderson KR, Abuhamad AZ, Ananth CV (2014). Prenatal detection of fetal growth restriction in newborns classified as small for gestational age: correlates and risk of neonatal morbidity. Am J Perinatol.

[13] Fratelli N, Valcamonico A, Prefumo F, Pagani G, Guarneri T, Frusca T (2013). Effects of antenatal recognition and follow-up on perinatal outcomes in small-for-gestational age infants delivered after 36 weeks. Acta Obstet Gynecol Scand.

[14] Lindqvist PG, Molin J (2005). Does antenatal identification of small-for-gestational age fetuses significantly improve their outcome?. Ultrasound Obstet Gynecol.

[15] O'Conner D. Saving Babies’ Lives: a care bundle for reducing stillbirth. NHS England; 2016. https://www.england.nhs.uk/wp-content/uploads/2016/03/saving-babies-lives-car-bundl.pdf.

[16] McCowan LM, Figueras F, Anderson NH (2018). Evidence-based national guidelines for the management of suspected fetal growth restriction: comparison, consensus, and controversy. Am J Obstet Gynecol.

[17] Clifford S, Giddings S, South M, Williams M, Gardosi J (2013). The Growth Assessment Protocol: a national programme to improve patient safety in maternity care. MIDIRS Midwifery Digest.

[18] Vieira MC, Relph S, Muruet-Gutierrez W, Elstad M, Coker B, et al. Evaluation of the Growth Assessment Protocol (GAP) for antenatal detection of small for gestational age: The DESiGN cluster randomised trial. PLOS Medicine. 2022;l19(6):e1004004. 10.1371/journal.pmed.1004004. 10.1371/journal.pmed.1004004 PMC9212153 35727800

[19] Anderson R (2008). New MRC guidance on evaluating complex interventions. BMJ.

[20] Hawe P, Shiell A, Riley T, Gold L (2004). Methods for exploring implementation variation and local context within a cluster randomised community intervention trial. J Epidemiol Community Health.

[21] Basch CE, Sliepcevich EM, Gold RS, Duncan DF, Kolbe LJ (1985). Avoiding type III errors in health education program evaluations: a case study. Health Educ Q.

[22] Vieira MC, Relph S, Copas A, Healey A, Coxon K, Alagna A, Briley A, Johnson M, Lawlor DA, Lees C (2019). The DESiGN trial (DEtection of Small for Gestational age Neonate), evaluating the effect of the Growth Assessment Protocol (GAP): study protocol for a randomised controlled trial. Trials.

[23] Pinnock H, Barwick M, Carpenter CR, Eldridge S, Grandes G, Griffiths CJ, Rycroft-Malone J, Meissner P, Murray E, Patel A (2017). Standards for reporting implementation studies (StaRI) statement. BMJ.

[24] Curran GM, Bauer M, Mittman B, Pyne JM, Stetler C (2012). Effectiveness-implementation hybrid designs: combining elements of clinical effectiveness and implementation research to enhance public health impact. Med Care.

[25] Landes S, McBain S, Curran G (2019). An introduction to effectiveness-implementation hybrid designs. Psychiatry Res.

[26] Moore G, Audrey S, Barker M, Bond L, Bonell C, Cooper C, Hardeman W, Moore L, O'Cathain A, Tinati T (2014). Process evaluation in complex public health intervention studies: the need for guidance. J Epidemiol Community Health.

[27] Damschroder LJ, Aron DC, Keith RE, Kirsh SR, Alexander JA, Lowery JC (2009). Fostering implementation of health services research findings into practice: a consolidated framework for advancing implementation science. Implement Sci.

[28] Steckler A, Linnan L (2002). Process Evaluation for Public Health Interventions and Research.

[29] Pfadenhauer LM, Gerhardus A, Mozygemba K, Lysdahl KB, Booth A, Hofmann B, Wahlster P, Polus S, Burns J, Brereton L (2017). Making sense of complexity in context and implementation: the context and implementation of complex interventions (CICI) framework. Implement Sci.

[30] Schellenberg J, Bobrova N, Avan B, London School of Hygiene and Tropical Medicine (2012). Measuring implementation strength: literature review draft report 2012.

[31] Proctor E, Silmere H, Raghavan R, Hovmand P, Aarons G, Bunger A, Griffey R, Hensley M (2011). Outcomes for implementation research: conceptual distinctions, measurement challenges, and research agenda. Adm Policy Ment Health.

[32] Gardosi J, Mongelli M, Wilcox M, Chang A (1995). An adjustable fetal weight standard. Ultrasound Obstet Gynecol.

[33] National Institute for Health and Clinical Excellence (2008). Antenatal care for uncomplicated pregnancies. NICE Clinical guidelines.

[34] Liamputtong P. Qualitative research methods. 4th ed. Australia and New Zealand: Oxford University Press; 2013.

[35] Saunders B, Sim J, Kingstone T, Baker S, Waterfield J, Bartlam B, Burroughs H, Jinks C (2018). Saturation in qualitative research: exploring its conceptualization and operationalization. Qual Quant.

[36] New ambition to halve rate of stillbirths and infant deaths [https://www.gov.uk/government/news/new-ambition-to-halve-rate-of-stillbirths-and-infant-deaths]. Accessed 9 Jan 2018.

[37] Robertson L, Knight H, Prosser Snelling E, Petch E, Knight M, Cameron A, Alfirevic Z (2017). Each baby counts: National quality improvement programme to reduce intrapartum-related deaths and brain injuries in term babies. Semin Fetal Neonatal Med.

[38] Evaluation of the implementation of the Saving Babies’ Lives care bundle in early adopter NHS Trusts in England (SPIRE). [https://cdn.ps.emap.com/wp-content/uploads/sites/3/2018/10/Saving-Babies-Lives-pilot-evaluation.pdf]

[39] Hugh O, Williams M, Turner S, Gardosi J (2021). Reduction of stillbirths in England from 2008 to 2017 according to uptake of the Growth Assessment Protocol: 10-year population-based cohort study. Ultrasound Obstet Gynecol.

[40] Lau YZ, Widdows K, Roberts SA, Khizar S, Stephen GL, Rauf S, Heazell AEP (2020). Assessment of the quality, content and perceived utility of local maternity guidelines in hospitals in England implementing the Saving Babies’ Lives care bundle to reduce stillbirth. BMJ Open Qual.

[41] Benning A, Ghaleb M, Suokas A, Dixon-Woods M, Dawson J, Barber N, Franklin BD, Girling A, Hemming K, Carmalt M (2011). Large scale organisational intervention to improve patient safety in four UK hospitals: mixed method evaluation. BMJ.

[42] McMullen H, Griffiths C, Leber W, Greenhalgh T (2015). Explaining high and low performers in complex intervention trials: a new model based on diffusion of innovations theory. Trials.

[43] Dixon-Woods M (2019). How to improve healthcare improvement—an essay by Mary Dixon-Woods. BMJ.

[44] Hargreaves JR, Goodman C, Davey C, Willey BA, Avan BI, Schellenberg JR (2016). Measuring implementation strength: lessons from the evaluation of public health strategies in low- and middle-income settings. Health Policy Plan.

[45] Craig P, Dieppe P, Macintyre S, Michie S, Nazareth I, Petticrew M (2008). Medical Research Council G: Developing and evaluating complex interventions: the new Medical Research Council guidance. BMJ.

[46] McInnes E, Dale S, Craig L, Phillips R, Fasugba O, Schadewaldt V, Cheung NW, Cadilhac DA, Grimshaw JM, Levi C (2020). Process evaluation of an implementation trial to improve the triage, treatment and transfer of stroke patients in emergency departments (T3 trial): a qualitative study. Implement Sci.

[47] Vousden N, Lawley E, Seed PT, Gidiri MF, Charantimath U, Makonyola G, Brown A, Yadeta L, Best R, Chinkoyo S (2019). Exploring the effect of implementation and context on a stepped-wedge randomised controlled trial of a vital sign triage device in routine maternity care in low-resource settings. Implement Sci.

