# Supplementary material for: Effect of the Growth Assessment Protocol on the DEtection of Small for GestatioNal age fetus: process evaluation from the DESiGN cluster randomised trial
Source: Implement Sci. 2022 Sep 5;17:60. doi: 10.1186/s13012-022-01228-1 (PMC9446790; doi:10.1186/s13012-022-01228-1)
Supplement: Supplementary file 2 — Additional file 2. Description of practice in clusters allocated to standard care. [file 13012_2022_1228_MOESM2_ESM.docx]

## Additional File 2

|  | Recommendations of GAP | Practice in standard care sites, as compared to fetal growth screening as recommended by GAP |
| --- | --- | --- |
| Staff training | At least 75% of each staff group (midwives, sonographers and obstetric doctors) should be trained using both face-to-face and e-learning methods on topics such as the evidence behind fetal growth screening and GAP, a standardised method to measure fundal height and the components of the growth assessment protocol. | No specific training recommended on screening of fetal growth anomalies, in addition to that recommended by standard professional training for these professions. |
| Fetal growth screening amongst women at low risk of SGA | Standardised serial fundal height measurements plotted on a customised growth chart, starting from 26-28 weeks gestation. Not suitable if large fibroids, BMI>35, multiple pregnancy. | Variation amongst sites regarding expected gestation at which to commence fundal height measurements (e.g. 24 weeks, 25 weeks for nulliparous women, 28 weeks for multiparous women).  Two sites recommended that fundal heights be plotted onto a population fundal height chart. Two sites recommended use of McDonald’s rule (i.e. that fundal height measured in centimetres should be within 2-3 of gestation measured in weeks). Woemn with a fundal height outside of the expected range should be referred for a fetal growth scan.  Two sites did not have guidance on this.  One site recommended assessment of uterine artery Dopplers at the anomaly scan of even low risk women and then referral for fetal growth scans at 28 and 36 weeks if abnormal (mean pulsatility index (PI) >1.25 or total PI>2.5). |
| Indications to refer women at low risk of SGA for a fetal growth scan | If first fundal height measurement < 10th Centile or Static growth or Slow growth (sequential measurements not following the slope of the curve)  Excessive growth/clinical suspicion of polyhydramnios: curve crosses centiles in an upward direction.   Note: first plot above 90th centile is not an indication for a growth scan. | Women at two sites are referred for fetal growth scans if the fundal height measurement is 3cm above or below the gestational age measured in weeks.  Women at two sites are referred for fetal growth scans if the fundal height measured below the 10^th^ centile (or above the 90^th^/95^th^ centile) on the population fundal height chart. One site also recommends referral when fundal height measurements are static.  Two sites provide no guidance on referral of low risk women for fetal growth scans. |
| Defining women at high risk of SGA | (Women with one or more of the following risk factors are defined as high risk) 1. Maternal risk factors  Maternal age > 40y Smoker (any) Drug misuse 2. Previous Pregnancy History Previous SGA baby (<10th customised centile) Previous Stillbirth 3. Maternal Medical History Chronic Hypertension Diabetes Renal Impairment  Antiphospholipid syndrome 4. Unsuitable for monitoring by fundal height Large fibroids BMI > 35  5. Current pregnancy complications  (Early pregnancy) PAPP-A < 0.415 MoM  Fetal echogenic bowel  Multiple pregnancy (Late pregnancy) Severe pregnancy induced hypertension or pre-eclampsia  Unexplained antepartum haemorrhage Concerns related to growth measurements, as listed above. | Wide variation identified in risk factors used to identify women at high risk of SGA. One site had no guidance on this.  Agreement that women with hypertension, diabetes and pre-eclampsia were considered at high risk.  There were differences in the threshold used for PAPP-A (<0.4 multiples of median), a previous small baby (i.e. <2.5kg), smoking (>10/day only)  Additional risk factors considered included young age, underweight BMI, poor obstetric history (e.g. previous pre-eclampsia or placental abruption), alcohol abuse, inflammatory bowel disease, sickle cell disease, mild or moderate pregnancy-induced hypertension,  One guideline split risk factors into major and minor risk factors, recommending different guidance according to type of risk. Examples of minor risk factors were nulliparity, BMI 30-35, smoking 1-10/day, low fruit intake. Two other guidelines also stratified risk using results of the uterine artery Doppler pulsatility index. |
| Fetal growth screening amongst women at high risk of SGA | Women with risk factors for fetal growth restriction should be referred to a consultant obstetrician or fetal medicine specialist.  The consultant-led team will refer for serial assessment (3 weekly until delivery) of fetal weight and umbilical Doppler from 26-28 weeks until delivery.   EFWs plotted on customised charts.   These women will not require plotting of fundal height measurements while such a serial scanning protocol is being followed. | Scan protocols for women at high risk of SGA vary widely, but sites offer a minimum interval of 4 weeks for any indication (unless SGA is detected). Sites who offer uterine artery Dopplers use these to increase or reduce the frequency of serial fetal growth scans according to the results.  All sites assess fetal growth using population-based fetal growth reference charts e.g. Hadlock. |
| Reporting outcomes | The birthweight, gestational age and any antenatal diagnosis of SGA of every baby should be reported using the GAP software at the time of birth.  The uploaded data is then used to generate monthly reports for each site of their performance in terms of SGA and detection rates. | No sites recommend reporting of outcomes. |
| Missed case analysis | Use of a missed case analysis tool is encouraged to support clinical governance teams in investigating events that led to a missed antenatal diagnosis of SGA. | No sites recommended routine analysis of missed cases of SGA. |
