# Supplementary material for: Effect of the Growth Assessment Protocol on the DEtection of Small for GestatioNal age fetus: process evaluation from the DESiGN cluster randomised trial
Source: Implement Sci. 2022 Sep 5;17:60. doi: 10.1186/s13012-022-01228-1 (PMC9446790; doi:10.1186/s13012-022-01228-1)
Supplement: Supplementary file 3 — Additional file 3. TIDIER description of the intervention. [file 13012_2022_1228_MOESM3_ESM.docx]

## Additional File 3 - Description of the intervention using the TIDiER guidance

| **Item number** | **Item** | **Description** |
| --- | --- | --- |
|  | **BRIEF NAME** | |
| **1.** | Provide the name or a phrase that describes the intervention. | The GAP programme is a complex intervention for improved detection of SGA infants through risk stratification, serial fundal height or scans during second and third trimester and use of customized charts for assessment of fetal growth. |
|  | **WHY?** | |
| **2.** | Describe any rationale, theory, or goal of the elements essential to the intervention. | Designed to improve antenatal detection of the small-for-gestational-age neonate thereby reducing stillbirth related to fetal growth restriction. This intervention has been proposed given the context of the UK having a stillbirth rate that ranks poorly compared to other similar health economies. |
|  | **WHAT?** | |
| **3.** | Materials: Describe any physical or informational materials used in the intervention, including those provided to participants or used in intervention delivery or in training of intervention providers. Provide information on where the materials can be accessed (e.g. online appendix, URL). | - Staff training materials - E-learning module - Evidence-based risk assessment and management protocols - GAP software for generation of customised growth charts and calculation of birthweight centiles - Tools to audit missed cases - Perinatal Institute support - Materials generated by the research sites in disseminating the intervention to staff and patients. |
| **4.** | Procedures: Describe each of the procedures, activities, and/or processes used in the intervention, including any enabling or support activities. | - Baseline audit on rates of antenatal detection of SGA neonates - Train the Trainers course for senior staff members - A minimum of 75% staff in each staff group at each site to receive the face-to-face and e-learning training on the intervention. - Risk assessment of each woman at antenatal booking and throughout pregnancy - Generation of customised growth charts - Protocols for referral for additional fetal growth scans - Calculation of customised fetal weight centiles at birth - Missed case audit and review tool |
|  | **WHO PROVIDED?** | |
| **5.** | For each category of intervention provider (e.g. psychologist, nursing assistant), describe their expertise, background and any specific training given. | - The following staff receive the same e-learning and tailored face-to-face training (tailored to assist in the component of the intervention which they will provide):   - Health care assistants   - Antenatal and intrapartum midwives   - Antenatal sonographers   - Obstetric consultants, registrars, training-grade SHOs (including those in Foundation Year or GP training), GPs (at some sites). |
|  | **HOW?** | |
| **6.** | Describe the modes of delivery (e.g. face-to-face or by some other mechanism, such as internet or telephone) of the intervention and whether it was provided individually or in a group. | - Training of staff provided face-to-face in group settings (either by Perinatal Institute or by hospital trainers) or individually (by hospital trainers). - Training of staff includes individual e-learning online training. - Risk assessment of women at booking and ongoing assessment of fetal growth throughout pregnancy on an individual basis in a face-to-face scenario. |
|  | **WHERE?** | |
| **7.** | Describe the type(s) of location(s) where the intervention occurred, including any necessary infrastructure or relevant features. | - Hospital antenatal clinics - GP clinics - Community antenatal clinics - Birthing centres (obstetric/midwife-led).   Necessary infrastructure: access to the website on a computer, printing facilities. |
|  | **WHEN and HOW MUCH?** | |
| **8.** | Describe the number of times the intervention was delivered and over what period of time including the number of sessions, their schedule, and their duration, intensity or dose. | - Training delivered (both face-to-face and e-learning) once per year to a minimum of 75% of staff from each healthcare group (midwives, doctors, sonographers). - Risk assessment for each woman at antenatal booking and at all later antenatal appointments - For low risk women, symphyseal-fundal height measured and plotted onto customized chart every 2-3 weeks from 26-28 weeks until the end of pregnancy. - For high risk women, fetal growth ultrasound every 3 weeks from 26-28 weeks until the end of pregnancy. - For all women, single assessment of neonatal birthweight centile at birth. |
|  | **TAILORING?** | |
| **9.** | If the intervention was planned to be personalised, titrated or adapted, then describe what, why, when, and how. | The intervention to be tailored for women according to risk assessment (for risk of having a small-for-gestational age neonate) performed at antenatal booking.  No adaptations to the intervention are recommended for the trial. |
|  | **MODIFICATIONS?** | |
| **10.** | If the intervention was modified during the course of the study, describe the changes (what, why, when, and how). | No modifications to the intervention were recommended but it is recognised that individual cluster sites may modify the intervention for tailored application at their own sites. |
|  | **HOW WELL?** | |
| **11.** | Planned: If intervention adherence or fidelity was assessed, describe how and by whom, and if any strategies were used to maintain or improve fidelity, describe them. | As per the methods of this manuscript. |
| **12.** | Actual: If intervention adherence or fidelity was assessed, describe the extent to which the intervention was delivered as planned. | As per the results of this manuscript. |
