# Supplementary material for: Effect of the Growth Assessment Protocol on the DEtection of Small for GestatioNal age fetus: process evaluation from the DESiGN cluster randomised trial
Source: Implement Sci. 2022 Sep 5;17:60. doi: 10.1186/s13012-022-01228-1 (PMC9446790; doi:10.1186/s13012-022-01228-1)
Supplement: Supplementary file 4 — Additional file 4. Logic model. [file 13012_2022_1228_MOESM4_ESM.docx]

## Additional File 4 - Logic model for the GAP intervention and the process evaluation
