# Supplementary material for: Effect of the Growth Assessment Protocol on the DEtection of Small for GestatioNal age fetus: process evaluation from the DESiGN cluster randomised trial
Source: Implement Sci. 2022 Sep 5;17:60. doi: 10.1186/s13012-022-01228-1 (PMC9446790; doi:10.1186/s13012-022-01228-1)
Supplement: Supplementary file 5 — Additional file 5. Topic guides for interviews with clinical staff. [file 13012_2022_1228_MOESM5_ESM.docx]

## Additional File 5 - Topic guides for semi-structured interviews with clinical leads and frontline staff

##

## Topic guide for semi-structured interviews with GAP leads in implementing sites

Establish purpose of interview: ensure opportunity to read PIS and gain informed consent before commencing interview.

Establish practice for confidentiality of content (e.g., use of pseudonyms for participants/colleagues, and others mentioned, including Trust site/ obscuring of roles).

Clarify participants understanding of the term ‘GAP’ and specify that when we refer to this, we refer to the entire intervention (baseline audit, training, protocols, risk assessment, GROW charts, missed case audit).

***Context and preparation***

1. How did you/your Trust come to hear about the GAP approach?
2. What are your thoughts about these interventions? (Did you think there was a need for these; explore evidence/policy/political driver contexts)
3. Other issues; Context –priorities, politics, other things happening in the Trust at the same time, or in the outside world and affecting the Trust/maternity department

Issues around whether SGA or stillbirth are considered problematic here.

***Early implementation***

As you know, (your) Trust is an early implementer in the DESiGN trial and is in the process of or has introduced the GAP approach, which is designed to increase antenatal detection of SGA babies. We are interested to find out about your experience of how these changes have been implemented at your Trust/hospitals within your Trust.

1. Could you tell me, from your perspective, how the GAP programme has been implemented here?
   1. **Who is your GAP team** - do you have representatives from each area (clinicians, midwives, sonographers)?
   2. **Timeline and ‘go live’ date – when did you go live/when are you planning to go live?**
   3. **What activities, promotion materials, posters, meetings or other steps have been taken to raise awareness of the GAP protocols? Who has done these?**
   4. **Has this Trust made any additional policy changes since implementing GAP (i.e., policy/resources regarding other Stillbirth Care Bundle elements - smoking cessation, reduced fetal movements or fetal monitoring in labour?)**
   5. **Baseline audit**
      - Who is doing it?
      - How much time will take to finish it?
      - Should the audit be done retrospectively x prospectively?
      - If completed, do you know what the baseline SGA detection rate was here?
2. **Explore which staff have been trained in the new protocol and in the use of customised growth charts; when this started, and how it has been going, whether there have been any difficulties with staff training.**
   1. Train the trainer: provided by PI.
      - Invite comments on the usefulness of this training, sufficiency of materials provided, and ongoing support by PI
   2. Face-to-face training:
      - The PI requires Trusts to reach 75% face to face ‘front line’ staff training before GAP can be implemented; is this a useful target? What figure would you propose before implementing GAP? (if you were advising another Trust, for example).
      - Have you encountered barriers or facilitators in relation to reaching this target?
      - How is face to face training done?
      - How long does it take - time allocation?
      - Who is in charge of cascading the training?
      - Training GPs
   3. E-Learning
      - The PI required trusts to reach 75% e-learning before ‘go live’ and this was altered to 75% within a year of receiving initial training. What barriers and facilitators have you encountered in reaching this target?
      - How much time does it take to complete e-learning?
      - What arrangements has your Trust made for staff to undertake this training? (Is it mandatory? Is it done in staff’s paid time, or in paid study time, or own time?)
      - Is it useful? What are your views on the materials and information provided?

***Full Implementation***

1. **Risk assessment for SGA**
   1. In your trust, who does the initial risk assessment for SGA?
   2. What approach is used? (GAP/RCOG/Other, or variation on these)
   3. What decisions have been made around BMI, smoking, referral pathways/scan frequency?
   4. If risk factors are identified, are women referred to an obstetrician for review?
2. **Customised growth charts (generated when, by whom?)**
   1. Who generates the chart?
   2. When are the charts created?
   3. Are there enough clinics/ultrasonographers/appointments available?
   4. Does generating the chart lengthen appointment times?( If so, with whom, and by how much?)
      - If ‘yes’ - how is your Trust responding to this? What impact has it had on staff? i.e., longer clinics, less time for lunch, finishing late, seeing fewer patients in one clinic
   5. Have you rearranged any services in order to accommodate GAP?
   6. Has the protocol lead to new issues (such as following up patients who don’t attend appointments, or being asked for additional scans which are outside the protocol?)
   7. Who does the scan referrals - doctors or midwives?
   8. Any other resource and capacity issues affecting this Trust during implementation (appointment/clinic availability, USS, AL, training, etc)?
3. **Management strategies for suspected SGA (plot below line/abnormal growth trajectory)**
   1. Are you aware of any differences between the trust protocol and perinatal institute guidance?
   2. Are there plans to audit these differences and review?

***Reflection***

1. Sustainability: in your view, is there resource to continue with GAP after the implementation period ends?
2. Do you think that it will be possible to either continue with GAP, if the trial shows a benefit, or to return to the approach used previously, if the trial does not show benefit?

Is there anything else you would like to add? (Provide an opportunity for participants to discuss any issue in relation to GAP implementation not covered by questions).

Thank participant and invite any questions about research or what happens next.

## Topic guide for semi-structured interviews with frontline clinicians in implementing sites

Establish purpose of interview: ensure opportunity to read PIS and gain informed consent before commencing interview.

Establish practice for confidentiality of content (e.g., use of pseudonyms for participants/colleagues, and others mentioned, including Trust site/ obscuring of roles).

Clarify participants understanding of the term ‘GAP’ and specify that when we refer to this, we refer to the entire intervention (baseline audit, training, protocols, risk assessment, GROW charts, missed case audit).

***Context/Preparation:***

What are your thoughts about the GAP interventions? (Did you think there was a need for these; explore evidence/policy/political driver contexts)

Other issues; Context – priorities, politics, other things happening in the Trust at the same time, or in the outside world and affecting the Trust/maternity department

Issues around whether SGA or stillbirth are considered problematic here.

***Early implementation***

As you know, (your) Trust is an early implementer in the DESiGN trial and is in the process of or has introduced the GAP approach, which is designed to increase antenatal detection of SGA babies.

We are interested to find out about your experience of how these changes have been implemented at your Trust/hospitals within your Trust. Could you tell me, from your perspective, how the GAP programme has been implemented here?

1. **Who is your GAP team** - do you have representatives from each area (clinicians, midwives, sonographers)?
2. **How was awareness raised** amongst staff? Education about local protocols?
3. **Awareness of any additional policy changes since implementing GAP.** (i.e., policy/resources regarding other Stillbirth Care Bundle elements – smoking cessation, reduced fetal movements or fetal monitoring in labour?)
4. **Were you involved in conducting the baseline audit?**
   1. Who is doing it?
   2. How much time will take to finish it?
   3. Should the audit be done retrospectively x prospectively?
   4. If completed, do you know what the baseline SGA detection rate was here?
5. **Involvement in cascading training or had training cascaded?**
   1. Train the trainer: provided by PI.
      - Invite comments on the usefulness of this training, sufficiency of materials provided, and ongoing support by PI
   2. Face-to-face training:
      - The PI requires Trusts to reach 75% face to face ‘front line’ staff training before GAP can be implemented; is this a useful target?
      - Have you encountered barriers or facilitators in relation to reaching this target?
      - How is face to face training done?
      - How long does it take - time allocation?
   3. E-Learning
      - The PI required trusts to reach 75% e-learning before ‘go live’ and this was altered to 75% within a year of receiving initial training.
      - What barriers and facilitators have you encountered in reaching this target?
      - How much time does it take to complete e-learning?
      - What arrangements has your Trust made for staff to undertake this training? (Is it mandatory? Is it done in staff’s paid time, or in paid study time, or own time?)
      - Is it useful? What are your views on the materials and information provided?

***Full implementation***

1. **Risk assessment for SGA**
   1. In your trust, who does the initial risk assessment for SGA?
   2. What approach is used?
   3. Has your local protocol been adapted in any way? Thoughts about this.
   4. If risk factors are identified, are women referred to an obstetrician for review, or directly to scans?
2. **Customised growth charts (generated when, by whom?)**
   1. Who generates the chart?
   2. When are the charts created?
   3. How does your trust deal with third trimester late bookers/transfers of care?
   4. Are there enough clinics/ultrasonographers/appointments available?
   5. Does generating the chart lengthen appointment times? ( If so, with whom, and by how much?)
      - If ‘yes’ - how is your Trust responding to this? What impact has it had on staff? i.e., longer clinics, less time for lunch, finishing late, seeing fewer patients in one clinic
   6. Has the protocol lead to new issues (such as following up patients who don’t attend appointments, or being asked for additional scans which are outside the protocol?)
   7. Who does the scan referrals - doctors or midwives?
   8. If a midwife or doctor makes a scan referral, can they be confident that the scan they have asked for will be done?
   9. Any other resource and capacity issues affecting this Trust during implementation (appointment/clinic availability, USS, AL, training, etc.)?
3. **Management strategies for suspected SGA (plot below line/abnormal growth trajectory)**
   1. Are you aware of any differences between the trust protocol and perinatal institute guidance?
   2. If a midwife or doctor makes a scan referral, can they be confident that the scan they have asked for will be done?

***Reflection***

1. Do you think that it will be possible to either continue with GAP, if the trial shows a benefit, or to return to the approach used previously, if the trial does not show benefit?
   1. How easy would it be to return to previous standard practice? Unlearning knowledge/skills.
2. Is there anything else you would like to add? (Provide an opportunity for participants to discuss any issue in relation to GAP implementation not covered by questions).

Thank participant and invite any questions about research or what happens next.

## Topic guide for semi-structured interviews with GAP leads in non-implementing sites

Establish purpose of interview: ensure opportunity to read PIS and informed consent before commencing interview.

Clarify participants understanding of the term ‘GAP’ and specify that when we refer to this, we refer to the entire intervention (baseline audit, training, protocols, risk assessment, GROW charts, missed case audit).

Outline interview scope and establish ground rules for confidentiality of content (e.g., use of pseudonyms for participants/colleagues, and others mentioned, including Trust site/ obscuring of role titles).

***Context / preparation***

As you know, your Trust is part of the DESiGN trial, and is in the delayed implementation arm. DESiGN is a cluster RCT being conducted to explore whether the GAP approach improves AN detection of SGA babies

1. How did you/your Trust come to hear about the GAP approach? What are your thoughts about these interventions?
2. Did you think there was a need for these; explore evidence/policy/political driver contexts
3. What was the response here when you found out the Trust was in the delayed implementation group?
4. Other issues; Context –priorities, politics, other things happening in the Trust at the same time, or in the outside world and affecting the Trust/maternity department

Issues around whether SGA or stillbirth are problematic here.

***Current practice***

1. Can you tell me, from your perspective, what it has been like to be in the delayed arm of this study?
2. At the moment, what aspects of the Stillbirth Care bundle is your trust using in current routine care, and how are you approaching these targets?
   1. Reducing smoking in pregnancy
   2. Detecting fetal growth restriction
   3. Raising awareness of reduced fetal movement
   4. Improving effective fetal monitoring in labour
3. Do you have a Trust lead for the Stillbirth Care Bundle?
4. What is your Trust’s current routine practice for detection of SGA babies?
   1. Include any booking risk assessment (what approach is used? RCOG/local protocol?)
   2. How are midwives asked to assess fetal growth during routine care and at what points during pregnancy this happens?
   3. Training: Does any current mandatory Trust training cover detection of SGA babies? Are there any other ways (such as newsletters/in-house presentations) that awareness of SGA detection is raised within the Trust?

***Reflection***

1. When the DESiGN trial ends, does your Trust plan to implement GAP?
   1. If yes:
      - **Does your Trust have, or plan to have, a GAP team? If so** - do you plan to have representatives from each area (clinicians, midwives, sonographers)?
      - **Is there a plan to have a designated lead clinician to implement GAP**?
      - **Are there any current plans to instigate staff training from Perinatal Institute?**
      - **Are there anticipated resource issues? (staff time, ultrasound clinics, local issues)**
      - **Timeline - what is your current understanding of what this might be?**
   2. If no, or unsure: What are the considerations for your Trust in relation to implementing GAP?
      - Is your Trust planning an alternative?

Is there anything else you would like to add?

(Provide an opportunity for participants to discuss any issue in relation to current practice in SGA detection or anticipated GAP GROW implementation not covered by questions).

Thank participant and invite any questions about research or what happens next.
