# Supplementary material for: Effect of the Growth Assessment Protocol on the DEtection of Small for GestatioNal age fetus: process evaluation from the DESiGN cluster randomised trial
Source: Implement Sci. 2022 Sep 5;17:60. doi: 10.1186/s13012-022-01228-1 (PMC9446790; doi:10.1186/s13012-022-01228-1)
Supplement: Supplementary file 6 — Additional file 6. Detail of approach to rigour used in DESiGN process evaluation and qualitative data analysis. [file 13012_2022_1228_MOESM6_ESM.docx]

Additional File 6: Detail of approach to rigour^[[1]](#footnote-1)^ used in DESiGN process evaluation and qualitative data analysis.

| **Element of rigour** | **Approach to addressing rigour during data analysis** |
| --- | --- |
| Theoretical rigour | After reviewing relevant implementation literature, we selected the CICI framework^[[2]](#footnote-2)^ as a basis for examining implementation processes within the context of the provider organisations (see Figure 1). All CICI domains were included for comprehensiveness. |
| Procedural rigour | We used pre-published protocols for the overall methodology and develop an a-priori analysis plan for qualitative data analyses.  Both analysts completed the qualitative data analysis before the main trial results were known, ensuring awareness of findings did not bias interpretation. |
| Methodological rigour and inter-analyst reliability | Both analysts used the same N-Vivo database but worked separately on own sections of the data; this ensured analytical transparency.  We used NVivo ‘memo’ function to document coding decisions so there was consistency. For example: *‘Relationships with colleagues’ coded in ‘sociocultural/social networks’.*  Memos also contained reminders of how we applied CICI domains to the GAP intervention, and to make a note of any issues we each encountered when using the CICI framework to code data.  Where we felt the CICI Framework concepts did not reflect the data we identified, we discussed before agreeing to add new ‘sub-nodes’ and recorded this in memos. For example: *‘Difficult to code for leadership/taking a lead in relation to intervention. New sub code added under implementation agents/social or professional role’.*  We shared interim findings with the evaluation lead, and with the co-investigator group. |
| Triangulation | We used triangulation of data sources (interviewing clinicians & GAP leads from different professions); triangulation of methods (comparing quantitative notes review observations with qualitative interview data) and researcher triangulation (the two data analysts were from different professional disciplines and therefore brought different perspectives). |
| Reflexive rigour | Analysts were aware of own differing research and clinical backgrounds; we considered our own positions and assumptions alongside our detailed examination of the political context of the research.  We have reported multiple and sometimes contradictory perspectives amongst interview participants where these were found in the empirical data. |


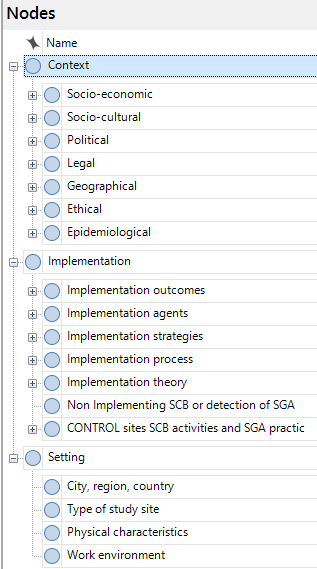


Figure 1 Initial nodes in NVivo12, using CICI Framework domains

1. Elements of rigour drawn from Liamputtong, P. and Ezzy, D. (2005) Qualitative Research Methods 2^nd^ Edition Oxford, Oxford University Press pp.38-55 [↑](#footnote-ref-1)
2. Pfadenhauer LM, Gerhardus A, Mozygemba K, et al. Making sense of complexity in context and implementation: the Context and Implementation of Complex Interventions (CICI) framework. Implement Sci 2017; 12(1): 21. [↑](#footnote-ref-2)
