# Supplementary material for: Effect of the Growth Assessment Protocol on the DEtection of Small for GestatioNal age fetus: process evaluation from the DESiGN cluster randomised trial
Source: Implement Sci. 2022 Sep 5;17:60. doi: 10.1186/s13012-022-01228-1 (PMC9446790; doi:10.1186/s13012-022-01228-1)
Supplement: Supplementary file 7 — Additional file 7. Supporting data on acceptability and feasibility. [file 13012_2022_1228_MOESM7_ESM.docx]

Additional File 7: Qualitative data related to ‘acceptability’ and ‘feasibility’ within ‘Implementation Outcomes’

| **Acceptability** (Based on Proctor et al’s definition: ‘the perception among implementation stakeholders that a given treatment, service, practice, or innovation is agreeable, palatable, or satisfactory’ | **Clinicians** | ***GAP leads*** |
| --- | --- | --- |
| **Acceptability of ‘GAP’ intervention** | *‘I believed GAP would be a good move towards detecting small babies and hopefully help any IUGR babies that we can detect and can prevent some intrauterine deaths. That would be a fantastic result if we can bring the numbers down nationally.’*  (HP93, Frontline staff, Site 9) | *‘I generally welcomed it, I was excited about it, I thought it was…a nice rigorous way of decision-making’.*  (SC21, GAP Lead, Site 10) |
|  | *“For me [GAP] is definitely very useful because if you take into consideration the ethnicity and the previous baby weight for each patient, I think you can be more specific and more accurate in evaluating the estimation of fetal weight”*  (HP1, Frontline Staff, Site 10) | *‘I think having, you know, a customised, standardised approach was really, really important so that then… at the point of training we could make sure that everyone was doing GAP recommendations so I think that was really, really good and just makes people really aware of …the rationale, you know..’*  (SC33 GAP Lead Site 7) |
|  | *‘…in the UK generally we have one of the highest still birth rates in Europe and because of that we want to try and implement this change and umm and therefore lower our stillbirth rates. But obviously that comes with difficulty because we are a country with a lot, with different populations across the country, different demographics and because of that we need to create a standardised system where we can we can screen for still – for SGA babies’*  (HP9, Frontline staff, Site 11) |  |
|  | *‘I think it’s a very good intervention. It will help us identify these small-for-dates babies and improve the outcomes’.*  (HP74, Frontline staff, Site 7) |  |
|  | *‘I found [Trust GAP training] extremely informative, very easy to understand and very logical and, to be quite honest, it’s something that I think trust wide, UK wide really, until something else comes about, everybody should be using them.’*  (HP52, Frontline staff, Site 11) |  |
|  | *It’s a lot better now because we all know the way we need to do it. We may not know the whole process behind GAP, but we know what we need to do and we think we are doing it right.*  (HP12, Frontline staff, Site 11) |  |
| **Acceptability: data which indicate *ambivalence* about GAP intervention** | *‘Generally, I think the majority of us don’t really want to [implement GAP]. We don’t really understand why we are doing it.…’ We don’t see the huge benefit it is to patients and we just weren’t clear on it’.*  (HP12, Frontline staff, Site 11) | *‘I didn't welcome it. I was a bit sceptical of it and maybe this was influenced from speaking to some of my colleagues in that I was a bit sceptical of the idea of needing to have a personalised growth chart.’*  (SC31, GAP Lead, Site 9). |
|  | *‘I think it is important to track each individual baby’s growth. I’m probably not convinced that one chart is better than another or that one protocol has been demonstrated to be better than another. I very much suspect it depends on the outcome you want to prevent. So I think there’s two different questions. Stillbirth is one but growth restriction is another and I think sometimes we think that fetal growth is the only cause of stillbirth. So I think if you are trying to reduce stillbirth you need to do more, but if you are just trying to catch growth restriction then the charts may be a good place to start.’*  (HP48, Frontline staff, Site 10) | *‘I clearly think now that…scientifically this approach [referring to customisation of growth charts] is...not evidence-based, it’s fundamentally wrong’.*  (SC18 GAP Lead, Site 10) |
|  | *‘There was a great emphasis on… I remember there being a great emphasis on selling the point that even if you save one baby it’s worth implementing the system. And yes, those are the main things that I remembe’r.*  *Interviewer: Do you agree with that?*  *‘I agree with the principle that if you even save one more baby than you would ordinarily then yes it’s well worth it. I don’t know whether I believe that it’s true’.*  (HP27, Frontline Staff, Site 11) | *‘We knew of the principle of GAP. I can't quite remember the timescales of everything, but there was the college guideline [whispers..] that we didn’t like [laughter] and which one of the registrars audited and we didn’t really do anything further other than auditing the potential impact of adopting what looked like a very complicated college guideline, and then there was the Perinatal Institute’s GAP GROW, and our commissioners were very keen. At the time trusts around here, some were using it, some weren’t, so there were mixed views’.*  (SC04 and SC07, GAP leads Site 8) |
|  | *‘I’ve had a little look at the evidence, I know there is some controversy because there’s not as yet been an RCT comparing the customised with population and some of the more… I know the studies that have been performed so far, some of them show that population might be better at picking up SGA babies, whether others show that customised might be better and it’s difficult because a lot of the data looking at the customised charts so far have come from the same group which in itself can create some bias. So it is a difficult thing and that’s why I think it’s good that we do have the DESiGN trial and it’s good that we’ve at last got what should be eventually an RCT comparing use of customised compared with population charts…because hopefully that will ultimately give us the ultimate answer as to which one is the better chart to use’.*  (HP26, Frontline staff, Site 10) | *‘I know that our [clinical service manager] was not keen to take part in the trial…because [they] did not believe that it would make any difference, and [they] didn’t want to spend any money, and it cost money’.*  (SC06, GAP Lead, Site 9) |
|  |  | *‘Then at some point I knew that [NHS Tust] was part of the trial, and then we were to implement the GAP trial. And I was sort of happy. I just didn’t know whether I’m a fan of customisation, but I’m not a fan of population charts either, so I was happy to be part of this to see whether it really works or not. Time will tell.’*  (SC05, GAP lead, Site 10) |
|  |  |  |
| Acceptability: ease of use and perceived effectiveness of GAP | *‘I think it’s quite simple really…I think it’s quite easy to plot’.*  (HP58, Frontline staff, Site 8) | *‘…actually, I was just getting some feedback today from staff and honestly, once they have got the hang of it, they can generate a chart in a couple of minutes now so it is not adding a great deal of extra work’.*  (SC17, GAP Lead, Site 11) |
|  | *‘I think [it is] better to use the customised growth chart because the rate of our intervention on small babies would be less’.*  (HP1, Frontline staff, Site 10) |  |
| Acceptability of GAP training | *‘I thought [face to face training] was good actually because it was… just quite engaging and…the information was presented in quite a clear manner as well as the evidence….I think [GAP] has had a good effect in that I think just having customised charts in place means that maybe when we are performing scans and when we do plot on the customised chart, maybe we are more likely to pick up the babies who are measuring tenth centile and below’*  (HP26, Frontline staff, Site 10) |  |
|  | *It was quite a while ago that I did [the e-learning]. I remember it with learning interesting facts. So I thought there was good theory in it that made sense to me and shone light on things that I saw as a clinician and that’s always nice, isn’t it – oh okay, that’s how that works. So I thought it was an interesting mix of theory and practical.*  (HP37, Frontline staff, Site 7) |  |
| Acceptability: Concerns about plotting errors and GAP identification of possible LGA babies without a corresponding care pathway | *‘I’d say probably half of the women are coming above the line and I’ve asked a couple of other [colleagues] and they seem to think theirs are coming up high... I just think a lot of women come up quite high on the chart and it can be quite worrying for them’.*  (HP91, Frontline staff, Site 10) | *‘I see so much erratic plotting in the charts… today they’re on the 90th centile and then three weeks later they are on the 10th centile, and they keep saying, “Yes it’s the same person”…So, four weeks ago the midwife thought that the baby was big, requested a scan; four weeks later the midwife (which could be a different one) thinks that the baby is small. And it’s all because of the chart.*  (SC05, GAP lead, Site 10) |
|  | *‘I’ve not seen so many babies charting below the line. A lot of them are actually charted as large-for-dates, we are getting quite a lot of that which is interesting’.*  (HP71, Frontline staff, Site 7) | *‘The other problem that the GAP chart has is that it then… it is designed to screen for small babies, but inadvertently, you end up finding large babies, so that has two implications. First of all, it creates a lot of anxiety for the woman. Secondly, it creates a lot of extra scanning for what will result in normal-sized babies with no clinical significance. And thirdly, you then have this other cohort of babies who are big on scan or above the ninetieth centile on scan, but that is not the point of the exercise, so it is more like an incidental finding that we found. Sometimes you scan babies that are plotting small and actually measure big on the scan and then you have to have that whole other conversation with the woman about this big baby, which you were not setting out to look for in the first place and you found a big baby and the uncertainties of the scan and whether it is actually a reliable screening test for a big baby and then the uncertainties you have about what we do about this big baby, so to cut a long story short, we have been inducing more women for supposed big babies because of the GAP programme.’*  (SC31, GAP Lead, Site 9) |
|  | *‘[GAP leads to identification of more large babies and therefore] lots of intervention that may not be warranted, it I mean it happens and also with the margin of error it’s not always accurate’*  (HP3, Frontline staff, Site 7) |  |
| **Feasibility** (Based on Proctor et al’s definition: ‘..the extent to which a new treatment, or an innovation, can be successfully used or carried out within a given agency or setting’ | ***Clinicians*** | ***GAP leads*** |
| **Feasibility – able to implement GAP** | *‘I just started generating the charts, assessing the women for who needs referral for scans straightaway or who just needs to have the chart generated. I found it quite straightforward, I thought it was quite self-explanatory. I did save a copy just of the flowchart of what to do.’*  (HP44, Frontline staff Site 9) |  |
| **Feasibility issues** | *‘The only problem we have got is that sometimes the growth chart is being missed for women…. we cannot store this growth chart in any of our computers and we cannot use, for example, the identification number to reprint the same growth chart’.*  (HP1, Frontline staff, Site 10). | *‘I have…appointments that I’ve sort of carved out, so 12 o’clock and 4 o’clock, to deal with...the ‘sudden’ reduced fetal movements or the ‘sudden’ patients being identified on GAP, that sort of thing. So we can try and always fit them in. But, I think a lot of ultrasound departments, they just leave the workflow to the receptionist to book in… and then the workload becomes unmanageable.*  (SC24, GAP lead, Site 11). |
|  | *‘…it has created quite a lot of work for us though. Obviously, ladies are having more scans, so they are coming to clinic more often, that makes the whole department a lot more busy’.*  (HP51, Frontline staff, Site 8) |  |
| **Feasibility: Training** | *‘In an ideal world, it would be great if everyone could do both [in person and online training]. In reality, in a health service that’s stretched and busy [laughs] it’s not always possible’.*  (HP23, Frontline staff, Site 11) | *‘I think the training really has to be facilitated. It’s… the online training is unnecessary’*  (SC18, GAP lead, Site 10)  *‘…you’ve got people that are very senior, and they had to take the [online training] test 10 times…there must be something there [laughs] that can be simplified’.*  (SC25, GAP lead, Site 9)  *‘I end up doing it [GAP e-learning] at home’.*  (SC05, GAP lead, Site 10) |
|  | *‘I can’t really remember [the e-learning]. I think it was okay. It is quite basic. I think I – not knew it already, but it was things I had already referred people for scans for. Some of it was confusing because it wasn’t applicable to our trust. I don’t think we have taken on board the entire recommendation. We have got our own modified version, so that was a bit, what’s the point?’*  (HP47, Frontline staff, Site 8) | *‘…there is not much money to train people or do still this research…and the midwives, they won’t come because they don’t have time off, so it’s a huge big struggle to train’.*  (SC28, GAP Lead Site 7) |
| **Feasibility: implementation** | *‘Yes, so I think in my team we are quite fortunate because we have got fairly good staffing. So the day before our clinic on a [weekday] we have a midwife set aside to do abnormal results, go through GAP charts and plan for the [next] clinic. So it’s all part of that day, but I know that other teams struggle a little bit to find that time to set aside.’*  (HP32, Frontline staff, Site 11) | *‘… implementing…it needed a full time role, I think… for it to [meet] the deadlines, and make sure everybody is on board, and, you know, bringing the staff with you, along with you’.*  (SC25 GAP Lead, Site 9) |
|  | *‘Unless there is an upward trajectory in all of the growth scans I’ve done, I’ve always queried it with people and in the instances that I’ve queried it I’ve usually had to go through at least one or two people until we get complete clarification on whether or not this person requires a growth scan’.*  (HP27, Frontline staff, Site 11) | *‘… we weren’t given any extra staff to implement this, we knew it was going to increase the number of scans, and yet we were expected to absorb that workload’.*  (SC19, GAP Lead, Site 9) |
| **Feasibility Resource** | *‘…the practical side is very difficult. We have a lot of patients who come and usually we are full, we are booked completely, and to fit the patient within three working days is very, very difficult. Sometimes we have to scan during our lunchtime which is not ideal at all but then otherwise we breach the time, we cannot fit them in three days and this is a real problem…’*  (HP41, Frontline staff, Site 9) | *‘Capacity for scanning, because we haven't got enough scan rooms. So it’s like the resource for your sonographers’.*  (SC04/SC07, GAP Leads, Site 8) |
|  | *‘That has been quite difficult. A lot of people didn’t know how to print a chart off. The IT access can be quite shoddy sometimes out in the community so it can be that somebody doesn’t have their chart printed at 16 weeks, then we see them at 28 weeks and someone forgets and doesn’t print’.*  (HP47, Frontline staff, Site 9) | *Interviewer: Have you needed to put on extra ultrasound clinics or anything…?*  *SC20: No. There’s no capacity for that… Because [the sonographers are] short-staffed anyway…And I think they worked out how much time it would take, and they would lose, I think they said 45 slots in a week, if they took on GAP. So they were, just very, um, unwilling.*  (SC20, GAP lead, Site 7) |
|  | *‘So when we go to a clinic…, we have to take the information from the computer, so blood results, scan results and document them in their…in their hospital notes which we take to the clinic and so if we have a clinic of 35/40 women, that is already a lot of um… documentation and then when we have to now produce a GAP chart that also involves, you know, extra time and we have to print it off as well as putting in the information so it is added work to our workload…’.*  (HP5, Frontline Staff, Site 11) | *‘…they said that if we implemented growth scans for all women who smoked within this Trust, they believe that our our ultrasound department would collapse under the weight. Um. So, that was why [our guideline] was changed, to be somebody smoking more than 10 cigarettes a day…’*  (SC06, GAP Lead, Site 9) |
|  | *‘And sometimes they are sent back and there are charts but our notes are horrendous that there is a GAP chart there, but it is just stuck randomly in their notes…so you will be told there is no GAP chart, but when you actually sit down and take time… you do find it, so again… yeah, they are not put consistently in an obvious place’.*  (HP7, Frontline Staff, Site 9) | *‘Er, [the GAP] BMI [referral point] is lower than ours, so we would only refer if they were 35 and over. Just because all of our women…we’d just be referring everyone’.*  (SC20, GAP lead, Site 7) |
|  | *Int: Okay. And roughly how many charts would you do in a single clinic?*  *HP71 ‘In my own clinic, a minimum of eight, but in the clinic, so when they come through… I work in the antenatal clinic. We get hundreds and hundreds of women coming through the door. I tried initially, when it started, to be doing them for every woman that came through the door. It just wasn’t feasible and this is where it went back to…[asking others to do this]’.*  (HP71, Frontline staff, Site 7) | *‘That’s one of the main issues around customisation. Generally speaking, you know, it is appalling how much, every time you want to do something new, it will require extra work, extra computers, extra IT, and these things cannot communicate[with each other]’.*  (SC18, GAP Lead, site 10) |
| **Feasibility: organisational software duplication, variance between GAP and /guidelines/protocol** | *‘So we are using [ultrasound generated charts] in conjunction with the GAP charts still because we still think that there is a place for them and because of our software package it would be impossible for us not to input that data. So at the moment, they are running alongside each other which at the beginning did generate some problems…’*  (HP23, Frontline staff, Site 11) | *‘…the [Trust] IT system doesn’t link in with the Perinatal Institute’s GAP GROW, which is possibly the case for a lot of people’s IT systems’.*  (SC04/SC07, GAP Leads, Site 8) |
|  | *‘Then we download the PDF [of the chart]. The only problem we have got is that sometimes the growth chart is being missed for women. The problem is that we cannot store this growth chart in any of our computers and we cannot use, for example, the identification number to reprint the same growth chart. We were trying to find a way with my senior colleagues to upload the specific growth chart in the program we use for ultrasound scans so like this we can keep it, but we are still wondering how to do it’.*  (HP1, Frontline staff, Site 10) | *The sonographers were very uncomfortable with not allowing the AC to drive the decision around further scanning….*  *…[they]…felt that they might get blamed if, you know, the EFW is normal but the AC is slightly dropping and they didn’t act accordingly. So what we have had to agree is that …if the sonographer’s still worried about the AC on the population chart…they can still go ahead and do a second scan in two weeks if they wish and do liquor volume, dopplers…but they must still plot the estimated fetal weight on the GROW chart without fail because I think they had kind of thought that they didn’t need to do it….”*  (SC17, GAP Lead, Site 11) |
|  | *‘So I think when GAP started we thought that it would increase the number of women that would need scanning, we thought it would put more pressure on the scan department, but if the chart is being used correctly, maybe not necessarily, I’m not sure. I think maybe we will need to look at what’s the impact for the sonographers since we started the GAP.’*  (HP78, Frontline staff, Site 7) | *‘We have had a big meeting not long ago, maybe less than two months – beginning of December, 10 December – to look at it because finding three-weekly slots is very difficult. Even if they are scan and go there is a likelihood that they could impact on the activity of sonographers definitely, but that has to be done as part of the protocol. Then the women themselves go, ‘I can’t come that week,’ and then it will become a fourth week.’*  (SC12/SC22, GAP Leads, Site 8) |
| **Feasibility; Solutions** | *‘…giving [staff] the time and the opportunity to do [online training]’.*  (HP23, Frontline staff, Site 11) | *‘I made sure that they all got dedicated time to do their e-learning. You know, not in the evenings, but I gave them a proper departmental time to do that..’*  (SC24, GAP Lead, Site 11) |
|  | *‘…So the day before our clinic …we have a midwife set aside to do abnormal results, so blood results, go through GAP charts and plan for the [extra] clinic…’.*  (HP32, Frontline staff, Site 11) | *‘One of our [doctors] did an audit…to see if we implemented the college guideline recommending the various parameters which we should be offering growth assessment for… We were looking at it and it became apparent that probably we are doing quite a good percentage of growth assessments, but whether they were targeted to the population which it should be targeted to, was the question raised. We did a feasibility… It did flag that we would require something like 100 hours extra…of sonography. That was discussed several times in various different [meetings] ..In fact, our sonography department…offered to train some additional capacity to create capacity – midwifery sonographers. They actually agreed to train and they themselves agreed to recruit. Lots of things were discussed beforehand and* ***that is why we were able to implement it quickly: because the groundwork was done before.***  (SC12/SC22, GAP Leads, Site 8) |
|  | *‘…before you transfer to postnatal ward…you have to complete…a trigger list of things – have you done this, have you done this, have you done this,* ***and they are getting IT to include*** *[birthweight/centile****] on there and I think that will…increase compliance’***  (HP37, Frontline staff, Site 7) |  |
|  | *‘I would normally use a ruler [for plotting] because I found it quite hard sometimes to see just with your eyes where you need to be plotting..’*  (HP44, Frontline staff, Site 9)  *‘And we actually brought set squares onto our training, onto the study days, to show the community midwives. And at the end of the training we did a quiz, whereby they were all given some charts to have a practise with and they could see how much easier using a set square actually made it. So hopefully most of them do have one’.*  (HP 46 & 58, Frontline staff, Site 8) |  |
|  | *‘So currently we are doing early morning scan lists to clear any backlog and we always have an urgent scan list, so an extra list five days a week which helps.’*  (HP71, Frontline staff, Site 7) |  |
|  |  |  |
