# Supplementary material for: Effect of the Growth Assessment Protocol on the DEtection of Small for GestatioNal age fetus: process evaluation from the DESiGN cluster randomised trial
Source: Implement Sci. 2022 Sep 5;17:60. doi: 10.1186/s13012-022-01228-1 (PMC9446790; doi:10.1186/s13012-022-01228-1)
Supplement: Supplementary file 8 — Additional file 8. Supporting data on context. [file 13012_2022_1228_MOESM8_ESM.docx]

| **Context and implementation process**  Favours implementation as intended  Impedes implementation as intended  I Impact on implementation unclear | | **Levels of context** | | |
| --- | --- | --- | --- | --- |
|  |  | **Micro (individual decisions or perspectives)** | **Meso (Trust/provider organisation level decisions/perspectives)** | **Macro (context external to provider organisation or individual)** |
| **Exploration and decision to adopt** | 1. Understanding the evidence for implementation   Context domain **Sociocultural** | Individual belief that GAP evidence is biased  Concern that population charts are not fit for purpose, outdated or discriminatory.  Concern that customised charts reflect racist discrimination and assumptions about normal fetal size/growth in given populations. | Staff groups disagree about evidence for GAP, or perceive evidence as uncertain  Ambivalent about evidence but agree important to do research, so willing to implement GAP for DESiGN trial participation. | National awareness of GAP as effective for reducing national stillbirth rate (media coverage, conferences).  Sonography training teaches that Fetal Abdominal Circumference is important for diagnose of SGA/FGR – GAP differs in this regard |
|  | 1. Concerns about the financial implications of GAP   Context Domain **Socio-economic** |  | Concern about shortage of sonographers/scan slots/clinic space/reliance on agency sonographers  Balancing urgency of scans between obstetrics and gynaecology depts  View that provider conducted unnecessary scans, and GAP could reduce this, or that provision of routine 3^rd^ trimester scans could be reviewed.  **Data from NON IMPLEMENTING SITES (which were randomised to intervention cluster) shows provider finance concerns influential in decision NOT to implement GAP (sites 12 and 13).** | National shortage of sonographers |
|  | 1. Desire for standardised care   Context domain **Political/health care system** |  | Aware current practice varies between consultants | MBRRACE reports show national stillbirth rates as high  RCOG ‘Each baby counts’ results |
|  | 1. Current practice perceived/ as problematic   **Context Domain Ethical/moral distress** | Morally distressing – staff individual experience of/reflection on feeling responsible for missed SGA cases with poor outcomes.  Some individual staff recognise low level of SGA detection from baseline audit.  Staff motivated to improve care quality and achieve better outcomes (4 sites). | Provider aware of past cases of SGA leading to poor outcomes/concerned about litigation costs  Provider perceives local stillbirth rates are too high.  Provider aware neighbouring hospitals using GAP and wants to give equivalent care  Staff view that interventions in response to reduced fetal movement (Saving Babies’ Lives care bundle) are already reducing stillbirth rate. | Secretary for State for Health ambition to halve stillbirths, Saving Babies’ Lives Care Bundle (awareness), need to comply with Clinical Negligence Scheme for Trusts(CNST) requirements (all sites)  NHS commissioners applying pressure to implement GAP (3 sites) |
|  | 1. Population requires specific support   **Context Domain**  **Epidemiology/demography** |  |  | Local population perceived not to engage with or have access to care (migrants, asylum seekers). |
| **Planning and preparation** | 1. Provider guidelines modified GAP   Context Domain **Legal/Guidelines** |  | View that Perinatal Institute recommendation of 3 weekly scans excessive |  |
|  | 1. Problems setting up GAP   Context Domain **Political/economic management** |  | Staff shortages meant one site was not initially able to identify a GAP lead midwife (1 site) |  |
|  | 1. Guideline revisions   Context Domain **Legal/Guidelines** |  | Delays whilst guidelines revised for GAP implementation (1 site) |  |
| **Initial Implementation** | 1. Arranging training   Context Domain **Political/economic management** | Staff discuss receiving training information via face to face meetings, emails, ‘cascades’ from GAP leads to managers. | Sites moved ‘face to face’ training into mandatory training days (4 sites)  High staff turnover meant that GAP training target not met (1 site)  Staff too busy to complete e-learning (4 sites)  Delay between initial GAP training and implementation problematic (1 site) |  |
|  | 1. Collaboration between staff   Context domain **Sociocultural** |  | Existing and supportive professional relationships meant that colleagues assisted each other with understanding new protocols (4 sites) |  |
| **Full Implementation** | 1. Impact of epidemiology on full implementation   Context domain  **Epidemiology** |  | Higher smoking and/or obesity rates in local population means need for scans is increased; not feasible to conduct this many scans.  Some providers scanned all women who smoke |  |
|  | 1. Impact of Geography on full implementation   Context domain **Geography** |  | High proportion of women attend provider from ‘out of area’ – centre is a tertiary referral unit, so demand for scans is higher. | Changes for GAP meant women have better access to scans, but may need to travel further |
|  | 1. Impact of Ethical considerations on full implementation   Context domain **Ethical** | Belief based on e-learning that GAP is clearly beneficial and will save babies  Staff concerned that GAP increases women’s anxieties, especially where scans suggest LGA and no care plan identified. (‘moral distress’)  Concern about unintended consequences - additional scans pick up other variations from normal (e.g. liquor volume, Doppler) and significance of these is unclear.  Concern that babies born with SGA identified by GAP do not receive tailored care in postnatal period (customised charts not implemented by paediatricians).  Staff unsure whether GAP will really ‘save babies’. | Professional **autonomy** over scan requests varies between sites – scan workload ‘managed’ by gatekeepers.  Concern that use of resource for GAP implementation might be better directed at other work to save babies’ lives. |  |
|  | 1. Impact of decision making and guidelines issues on full implementation   Context domain **Legal** | Belief that GAP improves accuracy of individual EFW measurement  Belief that GAP makes it easier to make decisions about scan referrals, and helps standardise care, and helps appropriately identify babies at need of further surveillance/intervention.  Inclusion of prompts and guidance on customised GAP chart helps inform referrals  Belief that GAP introduces uncertainty or is problematic to clinical decision making (e.g. AC not considered, focus on EFW)  Concern that GAP may increase Induction of labour; may be recommended on basis of scan, and difficult to discontinue once this has been mentioned even if subsequent growth appears normal.  Unclear what to do when GAP identifies possible LGA/large fetus  Deciding whether to refer for growth scan not straightforward, often need colleague/second opinion.  Anxiety about trying to reduce unnecessary scan referrals without missing abnormal growth.  Change to ‘usual practice’ over time confusing:  -scan if EFW <2.5kg or only if EFW>10^th^ centile?  -estimated SFH by palpation not comparable to scan measurements  -previous SGA is not well recognised risk factor for SGA in current pregnancy  -loss of ‘reassuring’ routine third trimester scan | Decision to move to printing customised GAP charts at 20-week appt (more time than at 12 week appt)  GAP protocol may be at odds with provider protocol embedded in provider software – leads to conflicting care plan.  Belief that scan referrals should be vetted to ensure ‘need’ for scan. |  |
|  | 1. Impact of politics, policies or influential people on service delivery during full implementation   Context domain **Political** |  | GAP timelines for scan referrals within 2-3 days ‘not realistic’; escalation policy ‘not always feasible’.  Provider doesn’t follow Perinatal Institute guideline with regards to frequency of scanning due to limitations in scan capacity  Executive board persuaded GAP/detection is important, so scan capacity increased but more still needed (one site).  Executive board aware of ‘breaches’ to scan times, willing to fund additional staff/resource  Provider ‘safety collaborative’ initiative also supported national Saving Babies’ Lives Care Bundle, helping to increase prioritisation of GAP  Senior leader (Director of Midwifery/Senior manager/lead obstetrician/Clinical Director provides extra appointments to support GAP  Problem of ‘transitioning’ to GAP guidance or aligning/mixing GAP and provider protocol guidance. Several sites report using elements of both GAP and own criteria for referrals.  Provider has ‘outside GAP’ protocols based on GAP but used when new pregnancy complications identified  Provider senior leader (e.g., Director of Midwifery/Senior manager/lead obstetrician/Clinical directors) voice opposition to GAP  E-learning mandatory but providers not always willing to pay for staff time to do this  No additional funding for sonographers  No additional capital funding for US machines | Awareness of ‘Saving Babies’ Lives’ care bundle has increased referrals from the community for suspected SGA  MBRRACE Reports  NHS 10-year plan  Regional network also focusing on stillbirth as a priority (alignment of focus supportive).  Neighbouring providers or ‘other hospitals’ implementing GAP  Jeremy Hunt (former Secretary of State for Health) Ambition to halve stillbirths in UK cited.  RCOG guidance ‘Every baby counts’ influential on practice  Being part of professional network (FIGO))  International Society of professionals in Ultrasound for Obstetrics and Gynaecology also has SGA detection guidance  GAPGROW protocol provides knowledge about differentiating between ‘normal’ small baby and ‘pathological’ small baby  GAP team and trainers regarded as experts; Jason Gardosi & Perinatal Institute receiving positive TV coverage  Kypros Nicolaides’ influence (Obstetrician and third trimester scan proponent)  Care Quality Commission visitor to provider advised against ‘rushing into’ GAP |
|  | 1. Impact of sociocultural knowledge and perceptions, or social capital on full implementation   Context domain **Sociocultural** | Observation that documenting previous SGA is helpful for identifying women at risk of SGA  GAP screening provides an ‘extra tool’ for screening women who don’t always attend for AN care, or have raised BMI, or are difficult to screen.  GAP training raised awareness of socio-economic inequality and risk of SGA  Staff feel a responsibility towards women to get detection right  Staff aware of/mention media coverage about stillbirth rates  Clinician came from country where GAP isn’t used – ‘totally new experience’  Clinician came from provider where GAP was used and was surprised to find it not in use here.  Belief that other providers using GAP have reduced stillbirth rates as a result  Aware from own training of variation in detection of SGA between providers/hospitals.  Before the DESiGN trial, unaware of GAP or Saving Babies’ Lives care bundle or research on reducing stillbirth (3 sites)  Staff describe helping each other out (multidisciplinary collaboration) to get scans done.  Staff describe encouraging colleagues to generate charts ‘it’s easy’  Belief that other providers using GAP have increased workload/scans/waiting times.  Provider staff do not think local or provider’s own stillbirth rates are higher than national average. | Improved recording [of SGA rates and scan referrals] makes it clear that Trust needs to invest more in specific areas (such as addressing high smoking rates). |  |
|  | 1. Impact of funding and socio-economic issues on full implementation.   Context domain **Socioeconomic** |  |  | Clinical Negligence Scheme for Trusts (CNST) 10% reduction if Trust (provider) is compliant with Saving Babies’ Lives care bundle initiatives |
| **Evaluation and reflection** | No new context data – covered already under ‘full implementation’ | | | |
| **Sustainment** | No new context data – covered already under ‘full implementation’, but concerns about unintended consequences such as possible increased intervention (Induction of Labour, pre-term birth); additional scans required and identifying other concerns including changed liquor volume or LGA fetus all discussed as issues affecting willingness to sustain intervention. | | | |

Standard Care Sites

| **Context data from clinical leads at sites randomised to ‘Standard Care’**  As the ‘standard care’ sites did not implement GAP, the data were coded under ‘exploration’ rather than in any of the later implementation process stages. | | **Levels of context** | | |
| --- | --- | --- | --- | --- |
|  |  | **Micro (individual decisions or perspectives)** | **Meso (Trust/provider organisation level decisions/perspectives)** | **Macro (context external to provider organisation or individual)** |
| **Exploration (in the context of recruitment to the DESiGN trial and potential to be randomised to either implement or to ‘Standard Care’)** | 1. Understanding the evidence for implementation   Context domain **Sociocultural** | Awareness of own uncertainty or scepticism about evidence for customisation, after attending conferences where the Intergrowth and customisation of growth assessment charts were discussed and debated.  Evidence that the GAP intervention is both cost/resource neutral and improves outcomes is needed prior to implementing GAP. | Clinical leads were aware that neighbouring sites would be introducing GAP as part of the DESiGN trial.  Perception that fellow leads at a regional network were supportive of gathering more information/evidence before widespread implementation of GAP.  Clinical leads discussed variation in SGA detection practices between their own organisations and other NHS organisations in England and in Europe, where other clinical tests are used, or ultrasound scanning in pregnancy is more frequent. Sense of being seen as ‘behind the times’ or providing inadequate services in relation to SGA detection and management. | Impression that customisation was being promoted through the Saving Babies’ Lives Care Bundle, RCOG 2013 Green top guideline.  Remembered that there was an expectation [at the time of recruitment to the DESiGN trial] that all Trusts would be required by NHS England to implement customisation of growth charts.  Publication of the ‘Intergrowth’ study (in 2014) led to uncertainties including a loss of faith in the need for the customised approach used within GAP.  External pressures on organisations (national campaign on stillbirth, Saving Babies’ Lives care bundle) lead to internal discussions about what each organisation was doing, but clinical leads were able to use own audit data to show that the organisation was already performing well. |
|  | 1. Concerns about the financial implications of GAP   Context Domain **Socio-economic** | The cost of implementing GAP perceived as small compared to the costs (emotional, social, economic) of failing to detect SGA or of stillbirth. | GAP perceived to have cost implications for ultrasound scanning, so having the DESiGN trial to evaluate clinical effectiveness of GAP rather than moving ahead to full implementation without RCT evidence considered important.  Organisation not thought likely to implement GAP (post-DESiGN trial) if the trial does not favour customisation, due to the costs of implementation. |  |
|  | 1. Desire for standardised care   Context domain **Political/health care system** | Sense of ‘doing nothing’ for duration of DESiGN trial problematic.  Sense that SGA management hasn’t had sufficient investment in terms of research or care provision or has been a low priority.  Delay to start of DESiGN trial experienced as problematic; difficult to make decisions about which care pathway to follow. | Trust boards asked clinical leads to explain how they would comply with the NHS England ‘Saving Babies Lives’ (SBL) care bundle whilst not implementing GAP.  Trusts had implemented ‘Saving Babies Lives’ care elements (Element 1 - reducing smoking in pregnancy, Element 2 - risk assessment and surveillance for fetal growth restriction, Element 3- raising awareness of reduced fetal movement, Element 4 effective fetal monitoring during labour).  Standard care sites did **not** use customised growth charts [this was a condition of being part of the DESiGN trial]; alternatives included fundal height measurement, some sites required that staff plot these measurements onto population based Symphysis-Fundal height chart.  View that Element 3 of SBL was leading to increased demand for ultrasound scans. |  |
|  | 1. Current practice perceived/ as problematic   **Context Domain Ethical/moral distress/change over time** | Perceived external pressure to reduce stillbirth by making changes in SGA detection and management, but also a belief that current practice was effective, and that this could be demonstrated through Trust clinical outcomes.  Concern that GAP would lead to increased ultrasound scanning of women at low risk of SGA or stillbirth, diverting resources from women who may be in more need of scant resources.  Use of numbers/centimetre estimates for symphysial fundal height perceived as outdated. | Being recruited into DESiGN and then randomised to ‘standard care’ meant organisations could modify own care pathways for SGA management and stillbirth prevention within the broader guidance of the SBL care bundle. |  |
|  | 1. Population demographic variations   **Context Domain**  **Epidemiological**  **Burden of disease** | View that GAP was not developed in a ‘normal’ population; the population [at standard care site] is different.  Perception that what organisation is doing already works well - already achieves lower stillbirth rates that national average. |  |  |
