# Supplementary material for: Effect of the Growth Assessment Protocol on the DEtection of Small for GestatioNal age fetus: process evaluation from the DESiGN cluster randomised trial
Source: Implement Sci. 2022 Sep 5;17:60. doi: 10.1186/s13012-022-01228-1 (PMC9446790; doi:10.1186/s13012-022-01228-1)
Supplement: Supplementary file 9 — Additional file 9. Characteristics of women included in the notes review. [file 13012_2022_1228_MOESM9_ESM.docx]

## Additional File 9 - Demographic characteristics of women and babies included in the notes review of implementation strength

| Characteristic | | Mean / Median / n | SD / IQR / % |
| --- | --- | --- | --- |
| Age at 12 weeks’ gestation | Age (mean/SD) | 31.2y | 5.6y |
|  | Women with age>40y at 12 weeks’ gestation (n/%) | 28 | 5.0% |
|  | Missing (n/%) | 39 | 6.6% |
| Ethnicity | United Kingdom (n/%) | 233 | 39.2% |
|  | Other European (n/%) | 114 | 19.3% |
|  | Middle East (n/%) | 23 | 3.9% |
|  | African (n/%) | 72 | 12.1% |
|  | Caribbean (n/%) | 15 | 2.5% |
|  | Asian (n/%) | 98 | 16.5% |
|  | North America (n/%) | 2 | 0.3% |
|  | South America (n/%) | 2 | 0.3% |
|  | Australian (n/%) | 1 | 0.2% |
|  | Mixed (n/%) | 16 | 2.7% |
|  | Unclassified (n/%) | 17 | 2.9% |
|  | Missing (n/%) | 2 | 0.3% |
| Body mass index | BMI (mean/SD) | 25.6 kg/m^2^ | 5.4 kg/m^2^ |
|  | BMI>35 (n/%) | 38 | 6.4% |
|  | Missing (n/%) | 0 | 0.0% |
| Parity | Median/range | 1 | 0-7 |
|  | Nulliparity (n/%) | 289 | 48.6% |
|  | Missing (n/%) | 1 | 0.17% |
| Birthweight | Median (IQR) | 3370g | 3025-3680g |
|  | Missing (n/%) | 4 | 0.7% |
|  | SGA by customised centile (n/%) | 80 | 13.4% |
| Gestational age at birth | Median (IQR) | 39^+5^ | 38^+5^ – 40^+4^ |
|  | Born preterm <37/40 (n/%) | 28 | 4.7% |
| Neonatal sex | Female (n/%) | 314 | 52.8% |
|  | Missing (n/%) | 2 | 0.3% |
