# Supplementary material for: Effect of the Growth Assessment Protocol on the DEtection of Small for GestatioNal age fetus: process evaluation from the DESiGN cluster randomised trial
Source: Implement Sci. 2022 Sep 5;17:60. doi: 10.1186/s13012-022-01228-1 (PMC9446790; doi:10.1186/s13012-022-01228-1)
Supplement: Supplementary file 10 — Additional file 10. Deviations of local guidelines from GAP recommendations. [file 13012_2022_1228_MOESM10_ESM.docx]

## Additional File 10 - Local deviations from guidance statements as recommended by GAP

| **Component of GAP affected** | **Summary of site deviations** |
| --- | --- |
| **Risk stratification** | **Re-categorises risk factors into new groups** e.g. major/intermediate/minor.  **Uses different definitions for high risk women:**  Smoker >10 rather than any smoker PAPP-A <0.3 or <0.4 rather than <0.415MoM Includes all women with PIH rather than just severe PIH  Uses ‘significant’ APH rather than ‘unexplained’ APH  **Groups missed of high risk group**  BMI 35-40  Drug misuse Previous stillbirth APLS Fetal echogenic bowel Unexplained APH  **Additional high risk groups used:**  Heavy bleeding 1st TM similar to menses  BMI<18 Low lying placenta GDM PPROM Fetal hydronephrosis Polyhydramnios >30cm Previous PET / early-onset PET Sickle cell disease  Previous SGA<10th centile on population chart Previous 2.5kg baby at term Single umbilical artery Current hyperthyroidism on medication New onset PIH (any severity) New GDM  **New intermediate risk category:**  For low-risk women with abnormal uterine artery Dopplers at anomaly scan. |
| Screening for low-risk women | Fundal height measurement recommended 2-4 weekly, rather than 2-3 weekly. |
| Screening for high-risk women | **Different frequency of growth scans:**  Varied with re-categorised risk status e.g. scans at 30 and 34 weeks’, or 28 and 36 weeks’, for moderate risk factors or scan at 34 weeks’ only if uterine artery Dopplers normal.  Recommends scans at 28 and 32 weeks’ only for women from some risk groups perceived as moderate risk e.g. smokers, drug users, high BMI or fibroids  Recommends scans at 28, 32 and 36 weeks’ for all high risk women (not 3-weekly as recommended)  Recommends 2-weekly scans for pre-eclamptic women only |
| Management of SGA | Also defines AC<5^th^ centile as being SGA and proceeds with management even if EFW >10^th^ centile. |
